# L-carnitine attenuated hyperuricemia-associated left ventricular remodeling through ameliorating cardiomyocytic lipid deposition

**DOI:** 10.3389/fphar.2023.1016633

**Published:** 2023-01-31

**Authors:** Yang Yang, Cuiting Lin, Qiang Zheng, Leqi Zhang, Yongmei Li, Qinghua Huang, Ting Wu, Zean Zhao, Lu Li, Jian Luo, Yanqing Jiang, Qun Zhang, Xing Wang, Chenglai Xia, Jianxin Pang

**Affiliations:** ^1^ Affiliated Foshan Maternity & Child Healthcare Hospital, Southern Medical University, Foshan, Guangdong, China; ^2^ School of Pharmaceutical Sciences, Southern Medical University, Guangzhou, Guangdong, China; ^3^ NMPA Key Laboratory for Research and Evaluation of Drug Metabolism & Guangdong Provincial Key Laboratory of New Drug Screening, School of Pharmaceutical Sciences, Southern Medical University, Guangzhou, Guangdong, China; ^4^ Good Clinical Practice Development, Guangdong Provincial Key Laboratory of Bone and Joint Degeneration Diseases, The Third Affiliated Hospital of Southern Medical University, Guangzhou, Guangdong, China

**Keywords:** hyperuricemia, cardiomyocytes, fatty acid, ventricular remodeling, L-carnitine

## Abstract

Hyperuricemia (HUA) is associated with left ventricular remodeling (LVR) and thereby causes the initiation and development of a large number of cardiovascular diseases. LVR is typically accompanied by cardiomyocyte energy metabolic disorder. The energy supply of cardiomyocytes is provided by glucose and fatty acid (FA) metabolism. Currently, the effect of HUA on cardiomyocytic FA metabolism is unclear. In this study, we demonstrate that UA-induced cardiomyocyte injury is associated with cytoplasmic lipid deposition, which can be ameliorated by the FA metabolism-promoting drug L-carnitine (LC). UA suppresses carnitine palmitoyl transferase 1B (CPT1B), thereby inhibiting FA transport into the mitochondrial inner matrix for elimination. LC intervention can ameliorate HUA-associated left ventricular anterior wall thickening in mice. This study showed that FA transport dysfunction plays is a critical mechanism in both cardiomyocytic injury and HUA-associated LVR and promoting cytoplasmic FA transportation through pharmacological treatment by LC is a valid strategy to attenuate HUA-associated LVR.

## 1 Introduction

Hyperuricemia (HUA) is regarded as a risk factor for various cardiovascular diseases (Zhang et al., 2019; Borghi et al., 2020). Clinical data have shown that for every 1 mg/dL increase in serum uric acid (UA), the odds of developing heart failure (HF) increase by 19%, and the risk of all-cause mortality and the composite endpoint in HF patients increased by 4% (Huang et al., 2014). Left ventricular remodeling (LVR) is the fundamental pathological process underlying the development of HF (Konstam et al., 2011). LVR is a continuous process involving responses to various types of injury to cardiomyocytes. Through LVR, the heart may temporarily preserve cardiac output, but progressive LVR is maladaptive and leads to HF (Pezel et al., 2021). The initial stage of LVR manifests as marked hypertrophy without ventricular enlargement or systolic functional impairment, followed by the development of chamber dilation and systolic dysfunction (Xia et al., 2009). The available studies have generally considered cardiomyocyte physiological activity with insufficient energy to be one of the mechanisms contributing to LVR development (Dalal and Mishra, 2017; Tuomainen and Tavi, 2017). Although optimizing energy metabolism therapy in patients with cardiovascular diseases is a feasible potential strategy, the underlying mechanisms of HUA-induced cardiomyocyte energy disorder are still unclear.

In adult hearts, glucose and fatty acid (FA) metabolism represents the major energy source to meet physiological demand. Energy metabolic homeostasis can be disrupted in response to many risk factors. These metabolic disorders include changes in glucose and FA transport from the extracellular environment into the cytoplasm, anaerobic glycolysis in the cytoplasm, and mitochondrial oxidative phosphorylation (Peterzan et al., 2017). Previous studies indicated that UA reduced glucose uptake in cardiomyocytes (Zhi et al., 2016; Jiao et al., 2021). However, the proportion of FA among energy substrates, was up to 50–70%, rather than glucose, indicating that FA dominate the cardiomyocyte energy metabolism (Lopaschuk et al., 2010). To date, few studies have investigated the influence of UA on cardiomyocytic FA metabolism. UA is a risk factor for cardiomyocyte injury, and the underlying mechanisms include insulin resistance, inflammation, and endoplasmic reticulum stress (Zhi et al., 2016; Yan et al., 2018; Zhang et al., 2020). These mechanisms can occur as a result of excessive cytoplasmic lipid-induced lipotoxicity (Lair et al., 2020; Lipke et al., 2022). The balance of cytoplasmic lipids is determined by both cell membrane-mediated FA uptake and mitochondrion-mediated elimination. We speculated that FA metabolic dysfunction may be the cause of UA-induced cardiomyocyte injury and thereby promote LVR.

We are interested in HUA-associated complications of multiple organ systems and the optimization of therapeutic strategies, and we explored potential pharmacological intervention approaches to attenuate the progression of HUA-associated complications based on the underlying pathological mechanisms. We previously established a procedure to induce human embryonic stem cells (hESCs) to differentiate into cardiomyocytes (Yang et al., 2019), which was used to explore the impact of UA on cardiomyocyte FA metabolism in this study. We previously also established a valid HUA animal model to explore urate-lowering therapy and organ protection (Chen et al., 2021; Li et al., 2021; Li et al., 2021; Lin et al., 2022), in this study this HUA animal model was used to observe the influence of pharmacological intervention with FA metabolism-targeting drugs on HUA-associated LVR and confirm the underlying mechanism. In this study, we show the role of FA metabolism in HUA-associated LVR. UA induced cardiomyocyte lipid deposition in the cytoplasm. FA β-oxidation facilitate drug L-carnitine (LC) ameliorate cardiomyocytic lipid deposition while β-oxidation inhibitor trimetazidine (TMZ) aggravated lipid deposition. UA stimulation suppressed CPT1B, which is the main factor involved in FA mitochondrial membrane transport. We further showed that LC intervention attenuated HUA-induced left ventricular wall thickening. This study showed that FA transport dysfunction plays is a critical mechanism in both cardiomyocytic injury and HUA-associated LVR and promoting cytoplasmic FA transportation into mitochondrion for metabolism through pharmacological treatment by LC is a valid strategy to attenuate HUA-associated LVR. It’s contribute to provide important pharmacological information for further optimization of therapeutic strategies.

## 2 Materials and methods

### 2.1 Cardiomyocyte generation and treatment

Cardiomyocytes were differentiated from hESCs (H1 cell line). Cardiac differentiation was performed as we previously described (Supplementary Figure 1A) (Yang et al., 2019). The cardiomyocytes showed characteristic cardiac troponin and actinin structures (Supplementary Figure 1B) and had high purity, as indicated by flow cytometry (Supplementary Figure 1C). The H1-derived cardiomyocytes were dissociated and seeded at a density of 3×10^4^ /cm^2^ on Matrigel-pretreated plates 2 days before pharmacological treatment. Cardiomyocytes were cultured with DMEM/F12 (Procell, PM150310) containing 10% FBS (Procell, 164210-50). UA (Aladdin, U105582), LC (Aladdin, C105423), and TMZ (Aladdin, T166941) were added to cells in the respective groups at the indicated concentrations.

### 2.2 Animal model and treatment

Healthy SPF male Kunming (KM) mice, weighing 20 ± 2 g, were provided by the Laboratory Animal Center of Southern Medical University (Guangzhou, China). All animal experiments were carried out following the guidelines of the Institutional Animal Care Committee of Southern Medical University and national animal experimental ethics standards. The animals were fed standard chow for 1 week in a suitable experimental environment with a 12-h/12-h light/dark cycle and a controlled temperature (25°C ± 1°C) and humidity (50 ± 10%). In early-stage HUA experiments, 32 mice were randomly divided into a blank group and a model group. The HUA group received daily intraperitoneal injection of potassium oxonate (PO) 350 mg/kg and oral gavage of hypoxanthine (HX) 450 mg/kg, while the blank group received the same amount of solvent. After 1 week of induction, all of the mice were sacrificed, and the blood was harvested for plasma UA determination, while the heart was harvested for real-time qPCR, western blotting, lipidomics analysis and immunofluorescence examination. For the pharmacological intervention in the chronic HUA experiment, 75 mice were randomly divided into five groups: the blank group, HUA model group, LC group, TMZ group and allopurinol (AP, Sigma, A8003) group. The LC group received 350 mg/kg LC, the TMZ group received 15 mg/kg TMZ, the AP group received 5 mg/kg AP gavage 1 h prior to modeling, and the other groups received saline. The modeling method was the same as that used in the short-term HUA experiment. 45 of the mice were sacrificed after 1 week intervention to quantify the molecules regulation in plasma and heart, and oil red O staining. The rest were continued experiment for 8 weeks of treatment and followed by echocardiography examination.

### 2.3 Cell viability analysis

The cell viability of cardiomyocytes was assessed with MTT assays. After pharmacological intervention in 96-well cell culture plates for 24, 48 or 72 h, the culture medium was removed and replaced with 0.1 mg MTT reagent (Beyotime, ST1537) per well dissociated in 100 μL basal DMEM (Procell, PM150210). Then, the cells were incubated at 37°C for 4 h, followed by replacement of the medium with 100 μL DMSO (Aladdin, D103272). After 10 min of shaking, viability was assessed by measuring the absorbance at a wavelength of 570 nm.

### 2.4 Oil red O staining

Oil Red O staining was used to examine cytoplasmic lipid deposition. The cells were fixed with 4% paraformaldehyde solution for 15 min, followed by washing with PBS buffer. Then, the fixed cells were dehydrated in 60% isopropyl alcohol for 10 min, stained with filtered Oil Red O solution (60% Oil Red O stock solution/40% water) for 20 min and rinsed with 60% isopropanol. Finally, the sections were counterstained with hematoxylin and then examined under a Zeiss microscope in bright field mode.

### 2.5 Reverse transcription and real-time quantitative PCR

Total RNA was extracted by using RNAiso Plus reagent (Takara, 9109) following the protocol recommended by the manufacturer. Reverse transcription was conducted using a StarScript II First-strand cDNA Synthesis Kit-II (GenStar, A214-10) following the protocol recommended by the manufacturer. Real-time qPCR was conducted in 96-well format plates using TB Green™ Premix Ex Taq™ (Takara, RR420A) following the protocol recommended by the manufacturer. For each analysis, the mRNA level was normalized to the levels of the housekeeping gene β-actin. Information on the primers used is presented in Supplementary Table 1.

### 2.6 Western blot analysis

Total proteins were lysed with RIPA buffer (Fude, FD009). Then, 20 μg of protein was separated on a 10% SDS–PAGE gel by electrophoresis, followed by transfer to a polyvinylidene difluoride membrane (Pall, BSP0161). Then, the membrane containing the protein was blocked with TBST buffer containing 5% skim milk powder for 1 h at room temperature. The membranes were incubated with primary antibodies at 4°C overnight. Then, the membranes were washed and incubated with the corresponding secondary antibodies for 2 h at room temperature. Signals were detected by using an ECL hypersensitive chemiluminescence kit (Boster, AR1191). Information on the antibodies used is listed in Supplementary Table 2.

### 2.7 ELISA analysis

The ELISA analysis was used to quantify the concentrations of particular molecules in plasma and heart tissues. ELISA analysis was performed with commercial ELISA kits following the protocol recommended by the manufacturer. The CPT1B quantification were performed by using mouse CPT1B ELISA kit (Meimian, MM-46720). The LC quantification were performed by using LC ELISA kit (Meimian, MM-95093O1). The epinephrine quantification were performed by using mouse epinephrine ELISA kit (Meimian, MM-0351). The noradrenaline quantification were performed by using mouse noradrenaline ELISA kit (Meimian, MM-0876).

### 2.8 Echocardiography examination

All of the animals underwent an echocardiography examination at the end of the 8^th^ week with an ultrahigh-frequency and high-resolution small animal ultrasonic imaging system (VEVO 2100, Visual Sonics). Before the ultrasound procedure, the mice were fasted for 8 h and intraperitoneally injected with 1.5% pentobarbital (45 mg/kg) 10 min prior to the procedure to anesthetize the animals. The left ventricular anterior wall (LVAW) and posterior wall (LVPW) thickness and the internal diameter (LVID) were measured in both the diastolic stage and systolic stage. The cardiac function parameters were calculated with software provided with the system based on the obtained measurements.

### 2.9 Histological and immunofluorescence examination

The animals were sacrificed, and the organs were isolated and transferred to Eppendorf tubes prefilled with 4% paraformaldehyde. Paraffin-embedded tissue specimens were used to prepare 4-μm-thick sections. For histopathological examination, the sections were subjected to Masson staining for routine procedures and then scanned with a Pannoramic Scanner in bright field mode. For immunofluorescence examination, after deparaffinization and rehydration, antigen retrieval with target retrieval solution was performed, and the sections were blocked and incubated with the respective primary antibodies overnight at 4°C. Then, the sections were washed with PBS and incubated with secondary antibodies for 1 h in the dark. Finally, the sections were stained with DAPI solution to visualize nuclei. Imaging of the heart sections was performed with a Pannoramic Scanner in fluorescence mode. The image scanning service was supported by Servicebio Technology Co., Ltd. (Wuhan, China).

### 2.10 Blood samples and biochemical analysis

Blood samples from all animals were collected in heparin anticoagulation tubes and then centrifuged at 3000 rcf for 10 min at 4°C. The supernatant was isolated and stored at −20°C for biochemical measurements. The UA concentration was measured with a QuantiChromTM Uric Acid Assay Kit (Cat. DIUA-250, BioAssay Systems, United States) according to the protocol recommended by the manufacturer.

### 2.11 RNA-seq analysis

The cell samples were harvested with RNAiso Plus reagent immediately after 24 h of treatment with blank medium or medium containing 15 mg/dL UA. Then, the samples were frozen immediately and stored at −80°C. The frozen samples were sent on dry ice to Majorbio Co., Ltd. (Shanghai, China), for sequencing. RNA-seq was performed using the Illumina NovaSeq 6000 platform. The generated raw reads were submitted to the NCBI sequence read archives (SRA) bearing accession number PRJNA846497.

### 2.12 Lipidomics analysis

The cell samples were harvested directly with a cell scraper after 24 h of treatment with precooled solution (methanol:acetonitrile:water = 2:2:1), followed by transfer into a 1.5 mL centrifuge tube. The mice heart were collected into a 2.0 mL centrifuge tube directly after 1 week modeling. Then, the samples were frozen immediately and stored at −80°C. The frozen samples were sent on dry ice to Majorbio Co., Ltd. (Shanghai, China), for lipidomics analysis. Lipidomic data have been deposited in China National GeneBank DataBase (CNGBdb) with accession code CNP0003906.

### 2.13 Quantification and statistical analysis

All of the bar charts and box plots were generated using GraphPad Prism 9. The bioinformatics figures were generated by using R3.4.2 software. Quantification of the immunoblots was performed with ImageJ. Two groups comparation were carried out *via* an unpaired two-tailed Student’s t-test. Multiple groups comparation were carried out *via* one-way analysis of variance with two-stage linear step-up procedure of Benjamini, Krieger and Yekutieli. P< 0.05 was considered to indicate statistical significance. Data significance was described as *p < 0.05, ** *p* < 0.01, *** *p* < 0.001.

## 3 Results

### 3.1 UA-induced cardiomyocyte injury is associated with excessive cytoplasmic lipids

**FIGURE 1:**
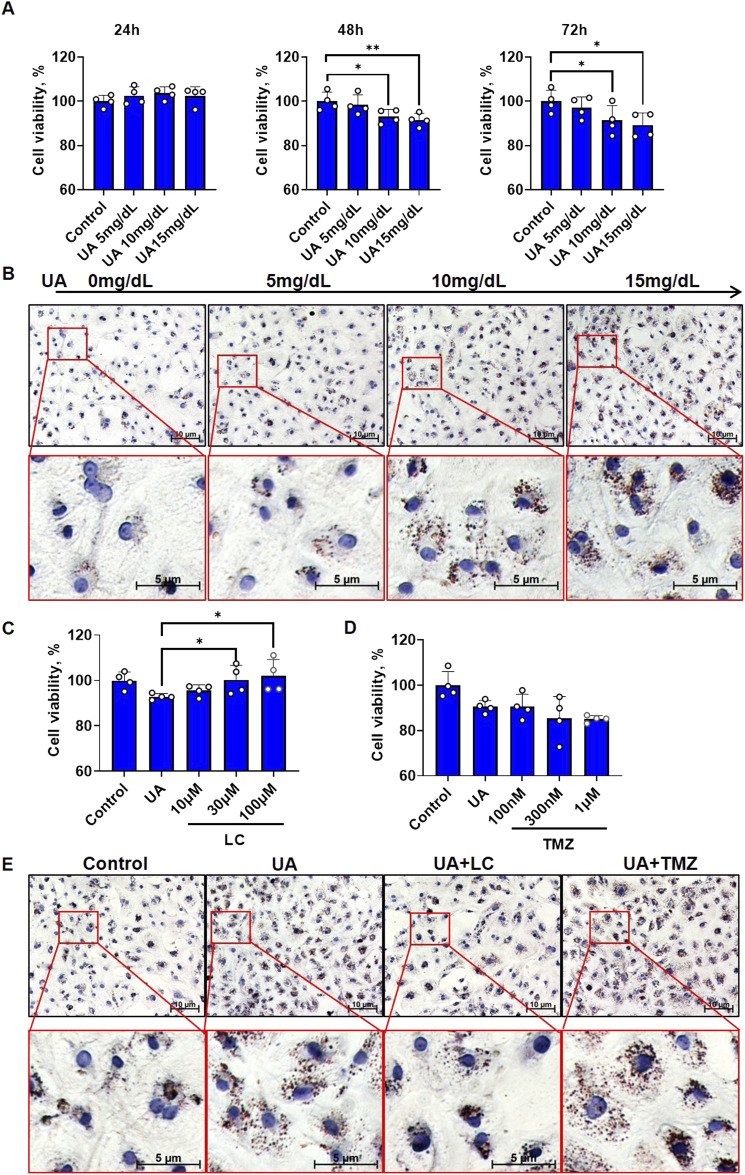
FA metabolic agents intervention affected UA induced cardiomyocyte injury. **(A)** The time-course of cell viability under different concentrations of UA exposure. The results were normalized to the mean value of control (*n* = 4); **(B)** Oil red O staining showing cardiomyocytic lipid deposition under different concentrations of UA exposure for 24 h. Dark red indicates deposited lipid, Blue indicated nuclei; **(C)** The influence of LC intervention on cell viability of cardiomyocytes under UA stimulation. UA concentration is 15 mg/dL. The cells were examined after 48 h treatment. The results were normalized to the mean value of control (*n* = 4); **(D)** The influence of TMZ intervention on cell viability of cardiomyocytes under UA stimulation. UA concentration is 15 mg/dL. The cells were examined after 48 h treatment. The results were normalized to the mean value of control (*n* = 4); **(E)** Oil red O staining showing UA induced cardiomyocytic lipid deposition under LC or TMZ intervention. UA concentration is 15 mg/dL, LC concentration is 100 μM, TMZ concentration is 1 μM. The cells were fixed for examination after 24 h treatment. Dark red indicates deposited lipid, Blue indicates nuclei.

Cardiomyocyte injury is considered to be a cause of LVR. We hypothesized that FA metabolic dysfunction may be the cause of UA-induced cardiomyocyte injury. First, we examined cell viability and cytoplasmic lipid changes under exposure to UA at various concentrations. The MTT assay showed that UA significantly decreased cardiomyocyte viability in a dose-dependent manner at both 48 h and 72 h (Figure 1A). This result is consistent with previous reports (Yan et al., 2018; Zhang et al., 2020). To confirm whether UA induced cardiomyocyte injury accompanied by FA metabolism, we examined cytoplasmic lipids by performing Oil Red O staining for visualization. The results showed that after 24 h of UA treatment, red staining, which represents deposited lipids, was also increased in a dose-dependent manner (Figure 1B; Supplementary Figure 1D).

To understand whether UA-induced cardiomyocyte injury is linked to excessive cytoplasmic lipids, two chemicals, LC, which promotes cytoplasmic FA oxidation, and TMZ, which inhibits cytoplasmic FA oxidation, were used to observe effects on cell viability. After cotreatment with UA for 48 h, LC ameliorated the UA-induced decrease in cardiomyocyte viability in a dose-dependent manner, with the strongest protective effect at 100 μM (Figure 1C), while TMZ did not show any protection and even exacerbated the UA-induced decrease in cardiomyocyte viability at a concentration of 1 μM (Figure 1D). Then, after cotreatment with UA for 24 h, Oil Red O staining was used to observe the effects of LC and TMZ on UA-induced lipid deposition. LC rescued UA-induced cytoplasmic lipid deposition, while TMZ exacerbated UA-induced cytoplasmic lipid deposition (Figure 1E; Supplementary Figure 1E).

### 3.2 UA-induced cytoplasmic lipid deposition is associated with suppression of CPT1B

**FIGURE 2:**
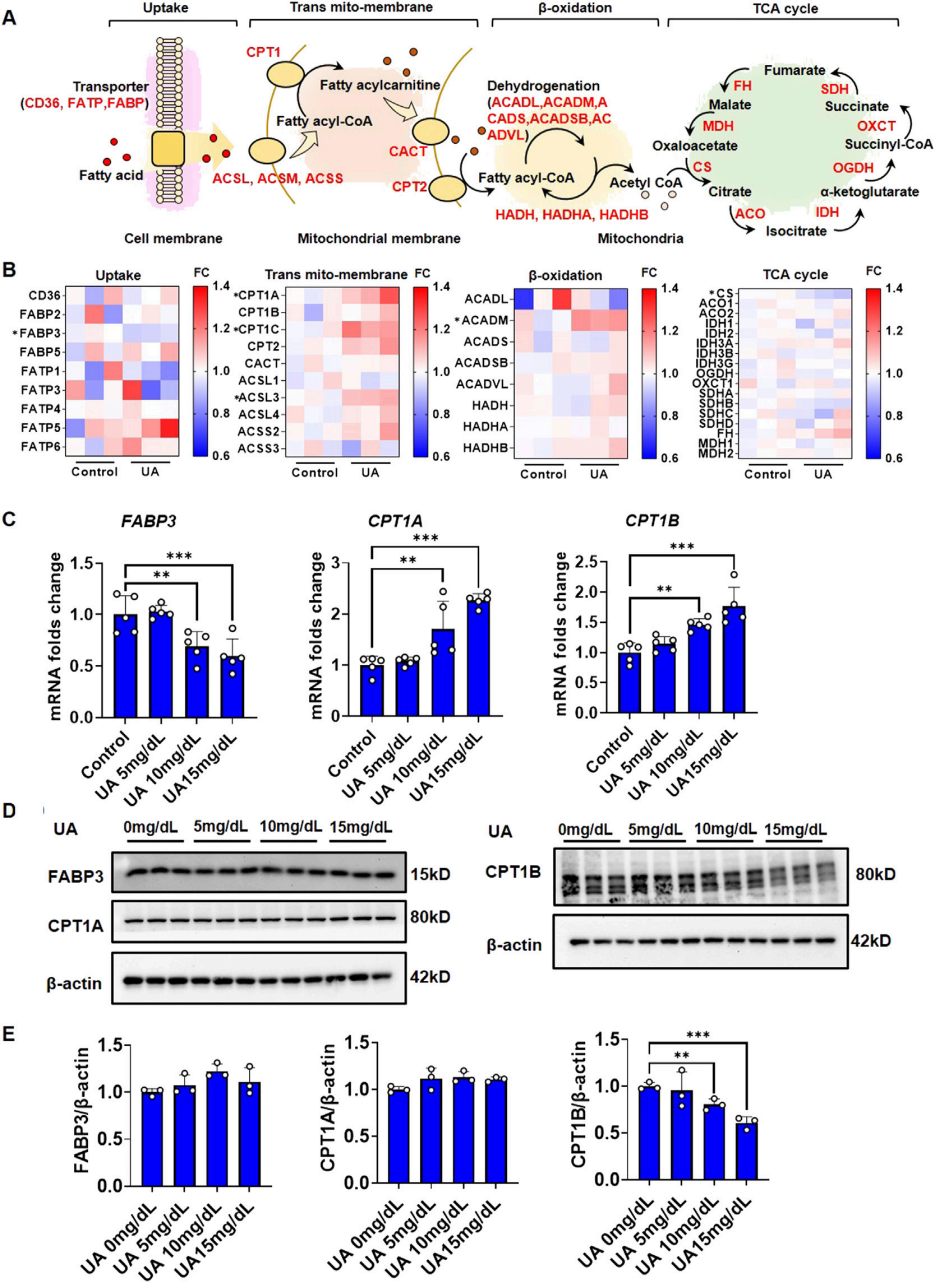
FA metabolic genes response under UA stimulation. **(A)** Diagram of steps and participated genes in fatty acid metabolism; **(B)** Heatmap reflect the expression fold change of fatty acid metabolic genes in each step. The expression data were generated from RNAseq; **(C)** Impact of UA on the gene expression fold change of FABP3, CPT1A and CPT1B. The cells were harvested after 24 h treatment followed by qPCR. The results were normalized to the mean value of control (*n* = 5); **(D)** Impact of UA on the protein level of FABP3, CPT1A and CPT1B. The cells were harvested after 1 h treatment followed by western blot analysis. β-actin was used as an internal loading control; **(E)** The quantification of Figure 2D.

The homeostasis of cytoplasmic lipids is maintained by both extracellular uptake and intracellular elimination. As shown in Figure 2A, free FA in the surrounding microenvironment enter cells *via* FA translocase (CD36), FA-binding protein (FABP), and FA-transport protein (FATP) on the cell membrane. In the outer mitochondrial membrane, FA are converted to fatty acyl-CoA by acyl-CoA synthases (ACSL, ACSM, and ACSS). Subsequently, fatty acyl-CoA is converted to fatty acylcarnitine by CPT1 catalysis and transferred to the inner mitochondrial membrane, and CPT2 regenerates fatty acyl-CoA from acylcarnitine. In the mitochondrial matrix, acyl-CoA dehydrogenases (ACADL, ACADM, ACADS, ACADSB, and ACADVL) and hydroxyacyl-CoA dehydrogenases (HADH, HADHA, and HADHB) catalyze fatty acyl-CoA β-oxidation to generate acetyl-CoA. Finally, acetyl-CoA is completely oxidized to carbon dioxide and water *via* the tricarboxylic acid cycle (TAC) to generate energy *via* a series of catalytic enzymes.

To identify the key steps and factors by which UA disrupts cardiomyocyte lipid homeostasis, RNA-seq was performed to elucidate the endogenous regulation of cardiomyocyte FA metabolism under UA stimulation for 24 h. A global transcription heatmap showed a strong gene expression profile for the RNA-seq data collected from cardiomyocytes with or without UA exposure (Supplementary Figure 2A). An MA plot diagram showed that after UA stimulation, a total of 1,408 genes were significantly upregulated, while 1716 genes were significantly downregulated (Supplementary Figure 2B). KEGG pathway enrichment analysis showed that the upregulated genes were associated with pathways that influence metabolism, such as insulin resistance and the AMPK signaling pathway, while the downregulated genes were associated with oxidative phosphorylation (Supplementary Figures 2C, D). RNAseq differential expression analysis showed that the expression of *FABP3, CPT1, ACSL3, ACADM*, and *CS* was significantly regulated by UA stimulation (Figure 2B). As known, the major cause of lipid deposition is FA uptake and β-oxidation. Therefore, the gene expression of significant differentially expressed genes in RNAseq were examined by qPCR. UA upregulated *CPT1A* and *CPT1B* expression while downregulating *FABP3* in a dose-dependent manner (Figure 2C). Gene expression regulation is the feedback to protein level changes. To identify the key protein that was directly influenced by UA, western blot analysis was performed. After UA exposure for 1 h, the protein level of CPT1B was significantly decreased in a dose-dependent manner, while FABP3 and CPT1A showed no significant differences (Figures 2D, E).

### 3.3 Lipidomics revealed that UA increased cardiomyocyte glycerolipids

Abnormal FA metabolism is the underlying cause of imbalances in cellular lipid composition. A decrease in the abundance of beneficial lipid species or an increase in the abundance of cytotoxic lipid species can induce lipotoxicity, thereby triggering a series of intracellular events, including insulin resistance and endoplasmic reticulum stress (Cao et al., 2011; Han and Kaufman, 2016). To further understand the impact of UA-induced FA metabolic dysfunction on cardiomyocytic lipid composition, cardiomyocytes treated with UA for 24 h and the corresponding control cells were subjected to untargeted lipidomic analysis to characterize lipid profile differences.

**FIGURE 3:**
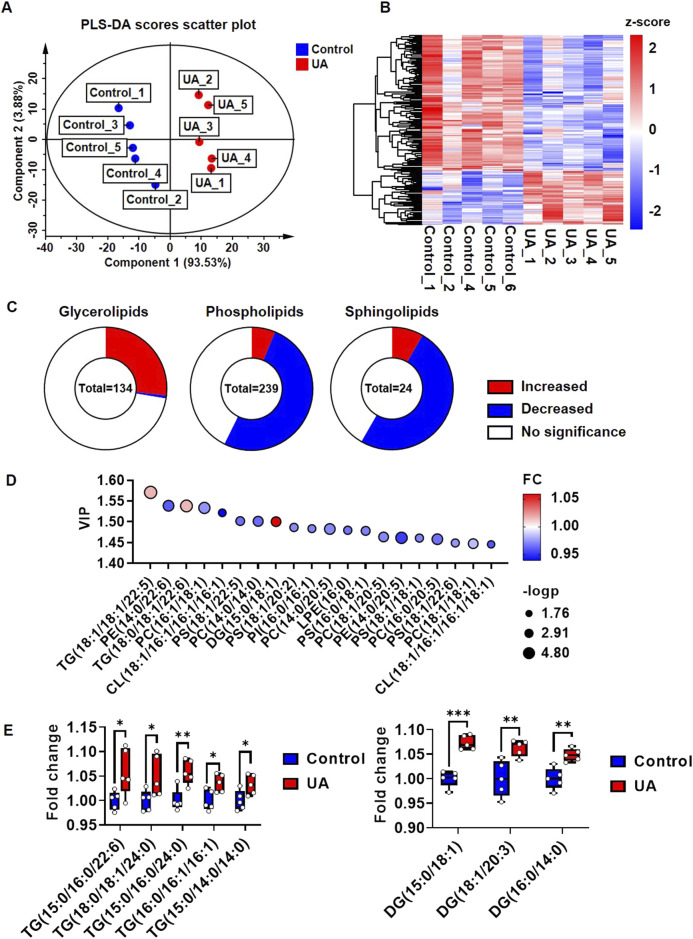
Lipidomic profile change of cardiomyocytes under UA stimulation. **(A)** PLS-DA score scatter plot showing the separation of control group and UA group. Cardiomyocytes were treated with 15 mg/dL UA. The cells were harvested for lipidomic analysis after 24 h treatment; **(B)** Heatmap showing the global view of the significant metabolites of control and UA treated cardiomyocytes; **(C)** Pie chart showing the changes in lipid composition under UA treated cardiomyocytes compare with control. P< 0.05 was considered as significance; **(D)** Bubble plots showing the top 20 significant metabolites based on VIP values; **(E)** Box plots showing the top five upregulated TGs and DGs. The results were normalized to the mean value of control (*n* = 5).

As shown in Supplementary Figure 3A, the lipomic analysis yielded 1,349 detectable metabolites. Among them, 961 metabolites were found in positive ion mode, and 388 metabolites were found in negative ion mode. In addition, 398 metabolites could be assigned to classes by comparison to the Human Metabolome Database (HDMB) (Supplementary Figure 3A). These 398 metabolites were classified as 239 glycerophospholipids, 134 glycerolipids, 24 sphingolipids and one fatty acyl lipid (Supplementary Figure 3B). In the PLS-DA score scatter plot, the lipidomic profiles of cardiomyocytes from the control and UA-treated groups were very different, and component 1 explained 93.53% of the variance (Figure 3A). R2Y and Q2 indicated the model’s goodness of fit (Supplementary Figure 3C). Among the 398 metabolites, 188 species showed significant differences in abundance (Figure 3B). Among the 134 glycerolipids, 36 species were significantly upregulated, while one species was downregulated. Among the 239 glycerophospholipids, 15 species were significantly upregulated, while 122 species were downregulated. Among the 24 sphingolipids, two species were significantly upregulated, while 12 species were downregulated (Figure 3C). Fatty acyl lipids showed no significant differences. To focus on the most important metabolites that contributed to the differences between control and UA-treated cardiomyocytes, only metabolites that passed the criteria of PLS-DA VIP score>1 were selected as discriminant metabolites (Figure 3D; Supplementary Table 3). The top five significant upregulated triglycerides (TGs) are TG (15:0/16:0/22:6), TG (18:0/18:1/24:0), TG (15:0/16:0/24:0), TG (16:0/16:1/16:1), TG (15:0/14:0/14:0). Only three diaglycerides (DGs) were significant upregulated, include DG (15:0/18:1), DG (18:1/20:3), DG (16:0/14:0) (Figure 3E). The analysis of the frequency in FA esterified TGs and DGs showed, 16:00, 18:00, 18:01 were top three highest frequency in upregulated TGs (Supplementary Figure 3D), while 18:01 was highest frequency in upregulated DGs (Supplementary Figure 3E).

### 3.4 Lipidomics revealed that HUA increased glycerolipids in mice hearts

**FIGURE 4:**
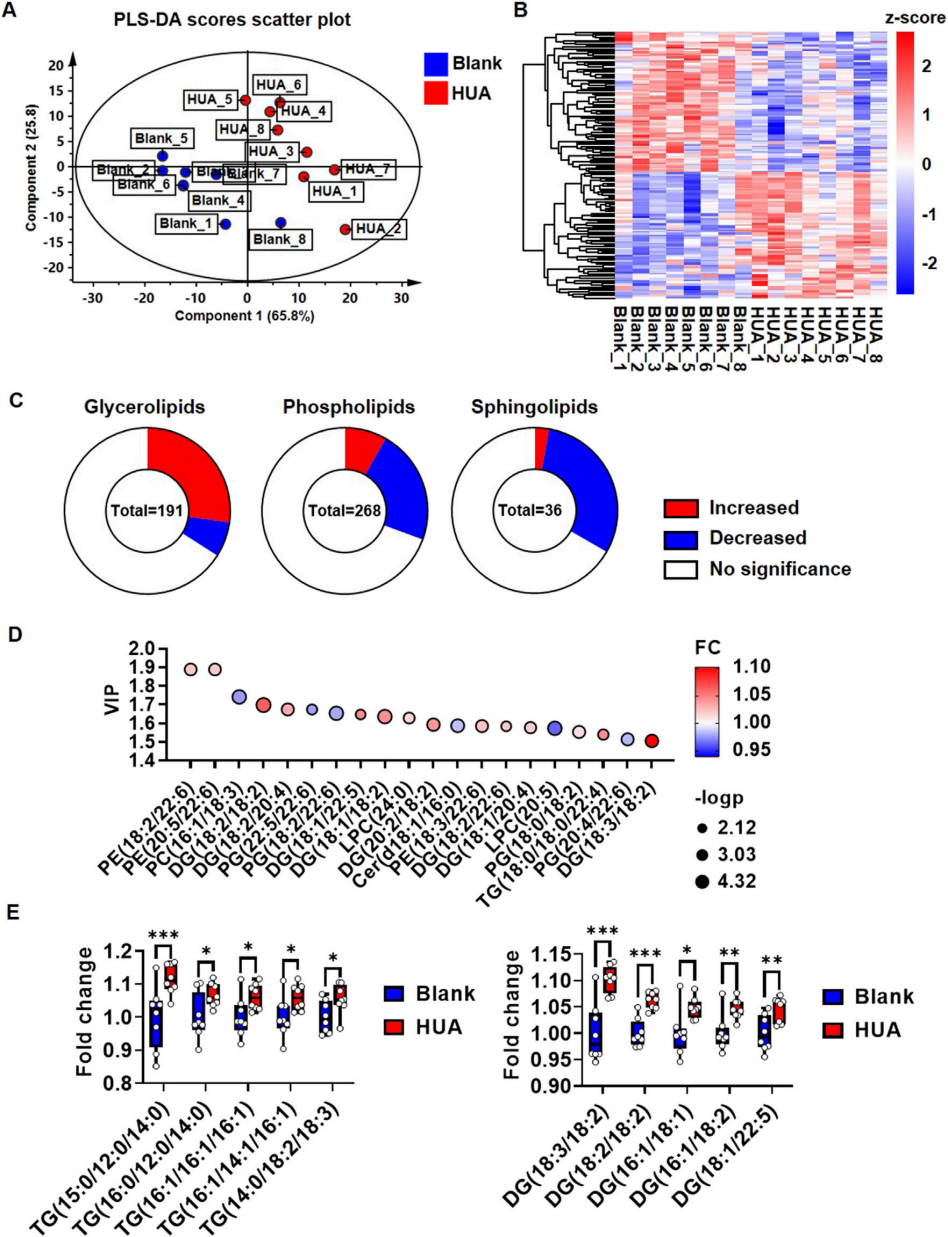
Lipidomic profile change of hearts under HUA induction. **(A)** PLS-DA score scatter plot showing the separation of blank group and HUA group; **(B)** Heatmap showing the global view of the significant metabolites of blank and HUA group; **(C)** Pie chart showing the changes in lipid composition of HUA compare with blank. P< 0.05 was considered as significance; **(D)** Bubble plots showing the top 20 significant metabolites based on VIP values; **(E)** Box plots showing the top five upregulated TGs and DGs. The results were normalized to the mean value of control (*n* = 8).

To confirm whether HUA influence *in vivo* cardiac lipid metabolism, a HUA mice model was constructed according to our previously publication (Lin et al., 2022). The heart tissues were harvested for analysis after 1 week HUA induction. The lipomic analysis of mice hearts yielded 1,368 detectable metabolites. Among them, 950 metabolites were found in positive ion mode, and 418 metabolites were found in negative ion mode. In addition, 496 metabolites could be assigned to classes by comparison to the HDMB (Supplementary Figure 4A). These 496 metabolites were classified as 268 glycerophospholipids, 191 glycerolipids, 36 sphingolipids and one fatty acyl lipid (Supplementary Figure 4B). The PLS-DA score scatter plot separated the blank and HUA groups well, and component 1 explained 65.8% while component 2 explained 25.8% of the variance (Figure 4A). R2Y and Q2 indicated the model’s goodness of fit (Supplementary Figure 4C). Among the 496 metabolites, 159 species showed significant differences in abundance (Figure 4B). Among the 191 glycerolipids, 52 species were significantly upregulated, while 13 species were downregulated. Among the 268 glycerophospholipids, 22 species were significantly upregulated, while 60 species were downregulated. Among the 36 sphingolipids, one species was significantly upregulated, while 11 species were downregulated (Figure 4C). Fatty acyl lipids showed no significant differences. The metabolites with the criteria of PLS-DA VIP score >1 were selected as discriminant metabolites and were shown in Figure 4D; Supplementary Table 4. The top five significant upregulated TGs are TG (15:0/12:0/14:0), TG (16:0/12:0/14:0), TG (16:1/16:1/16:1), TG (16:1/14:1/16:1) and TG (14:0/18:2/18:3). The top five significant upregulated DGs are DG (18:3/18:2), DG (18:2/18:2), DG (16:1/18:1), DG (16:1/18:2) and DG (18:1/22:5) (Figure 4E). The analysis of the frequency in FA esterified TGs and DGs showed, 16:00, 16:01, 18:01 were top three highest frequency in upregulated TGs (Supplementary Figure 4D), while 18:01, 18:02 were highest frequency in upregulated DGs (Supplementary Figure 4E). Furthermore, the oil red O staining showed it could be observed lipid droplets in some sections of HUA mice heart (Supplementary Figure 4F).

### 3.5 *In vivo* CPT1B suppression is associated with decreased endogenous LC

**FIGURE 5:**
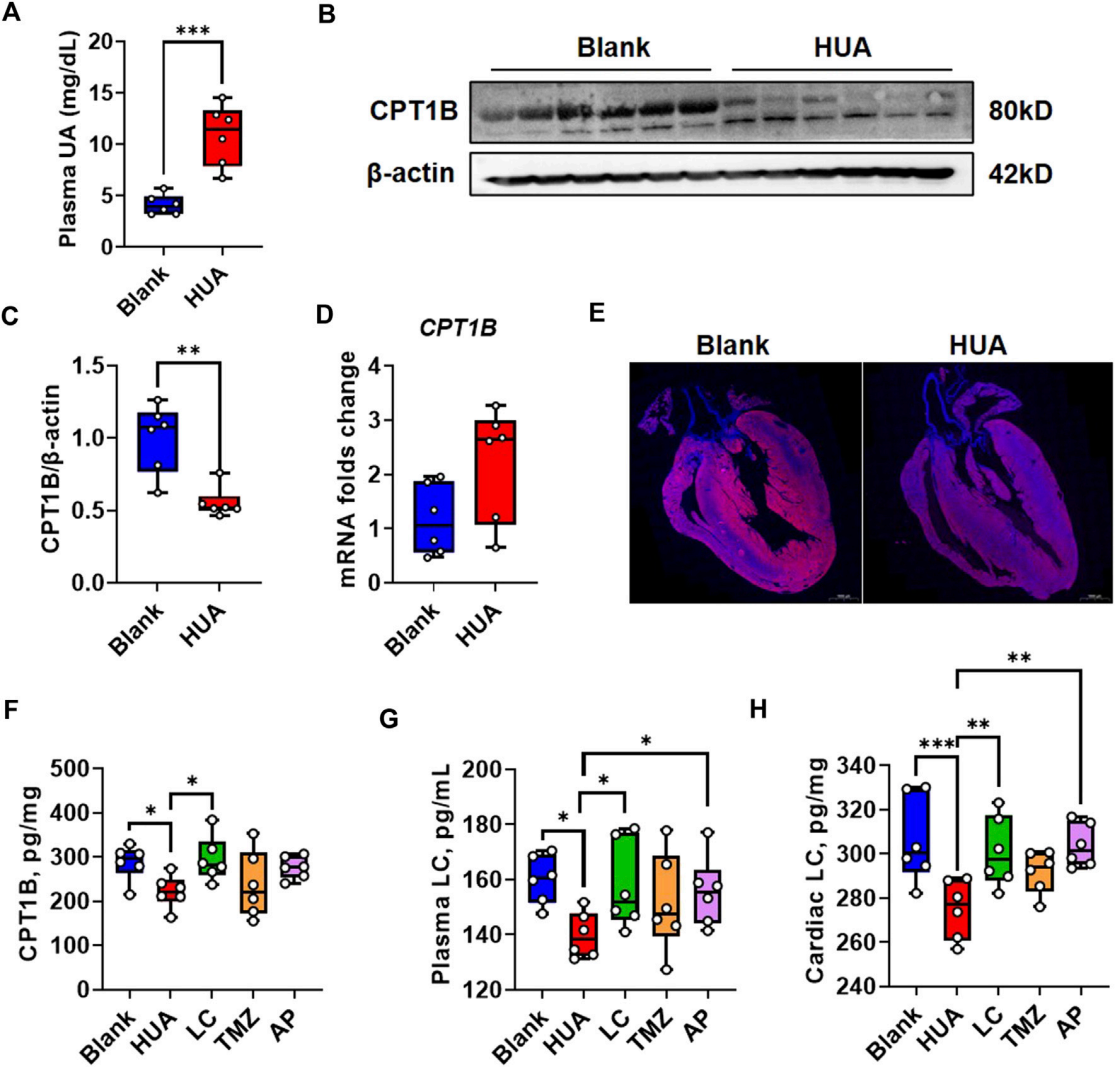
CPT1B response in early-stage HUA mice. **(A)** The UA concentration in plasma of blank and HUA group respectively (*n* = 6); **(B)** The protein level of CPT1B in the heart samples from blank and HUA group respectively, β-actin was used as an internal loading control; **(C)** The quantification of Figure 5B; **(D)** The mRNA folds change of CPT1B in the heart samples from blank and HUA group (n = 6); **(E)** The immunofluorescence reflects the distribution of CPT1B in the heart samples from blank and HUA group respectively. DAPI indicates nuclei, scale bar = 1000 μm; **(F)** The protein level of CPT1B in heart samples by ELISA quantification (n = 6); **(G)** The plasma LC concentrations by different treatment (*n* = 6); **(H)** The cardiac plasma LC concentrations by different treatment (*n* = 6).

We further examined the performance of CPT1B in heart tissues of HUA mice. The plasma UA examination indicated that the HUA model mice showed significantly increased plasma UA levels compared with those of the blank control mice (Figure 5A), which indicated the validity of the animal model. Consistently with the *in vitro* finding that the western blot examination showed the CPT1B protein level in HUA model group was significant suppressed compare with blank control (Figures 5B, C). It’s also consistently with *in vitro* experiment that the gene expression of *CPT1B* was upregulated in HUA model group compare with the blank group, but the differences showed no significance (Figure 5D). Subsequently, the whole hearts were performed immunofluorescence scanning and the result showed that CPT1B expression had a heterogeneous distribution. Cardiomyocytes close to the left ventricular endocardium showed higher CPT1B protein levels than cardiomyocytes in the inner field. Compared with those from the blank control group, the heart samples from the HUA group showed reduced CPT1B expression, especially in fields close to the endocardium (Figure 5E).

The *in vitro* experiment indicated UA induced cytoplasmic lipid deposition could be rescued by exogenous LC supplement while be exacerbated by TMZ. These two chemicals are both commonly used drugs in cardiovascular diseases. Therefore, the performance of these two drugs on CPT1B is worth to discuss. The CPT1B levels in heart tissues by different treatment were quantified by ELISA analysis, and the result indicated HUA induced CPT1B suppression could be rescued by LC treatment. However, the TMZ *in vivo* treatment was also showed some improvement on CPT1B but with no significance, which was not consistent with the *in vitro* result (Figure 5F).

The activity of CPT1 family is linked to the levels of endogenous LC (Saraiva et al., 2018). Therefore, we examined both concentrations of plasma LC and cardiac LC by ELISA quantification. The result showed that the both plasma and cardiac LC concentrations in HUA group were downregulated compare with blank group, which could be rescued by exogenous LC or AP. The performance of TMZ was not as predicted (Figures 5G, H). In current studies, the mechanisms involved in UA induced cardiomyocytic injury include ROS/CaMKIIδ/Parkin pathway (Gao et al., 2021), AMPK signaling pathways (Jiao et al., 2021), NLRP3 inflammasome (Zhang et al., 2020), and calpain-1 and endoplasmic reticulum stress (Yan et al., 2018). We constructed PPI network by using CPT1B and these previously published pathways to predict the role of CPT1B in UA induced signal cascades (Supplementary Figure 5A). The result indicated CPT1B is associated with UCP2 and SLC2A4, which reflected mitochondrial uncoupling and glucose uptaking (Supplementary Figure 5B).

### 3.6 LC ameliorates HUA-associated left ventricular anterior wall thickness

LVR is the pathological basis of the initiation and development of various heart diseases (Konstam et al., 2011; Pezel et al., 2021). LVR is manifested by increased wall thickness and diastolic dysfunction during earlier stages. We found that CPT1B suppression related FA metabolic dysfunction is a mechanism of UA induced cardiomyocytic injury and could be rescued by exogenous LC. Subsequently, we examined whether HUA associated LVR could be ameliorated by LC pharmacological intervention.

**FIGURE 6:**
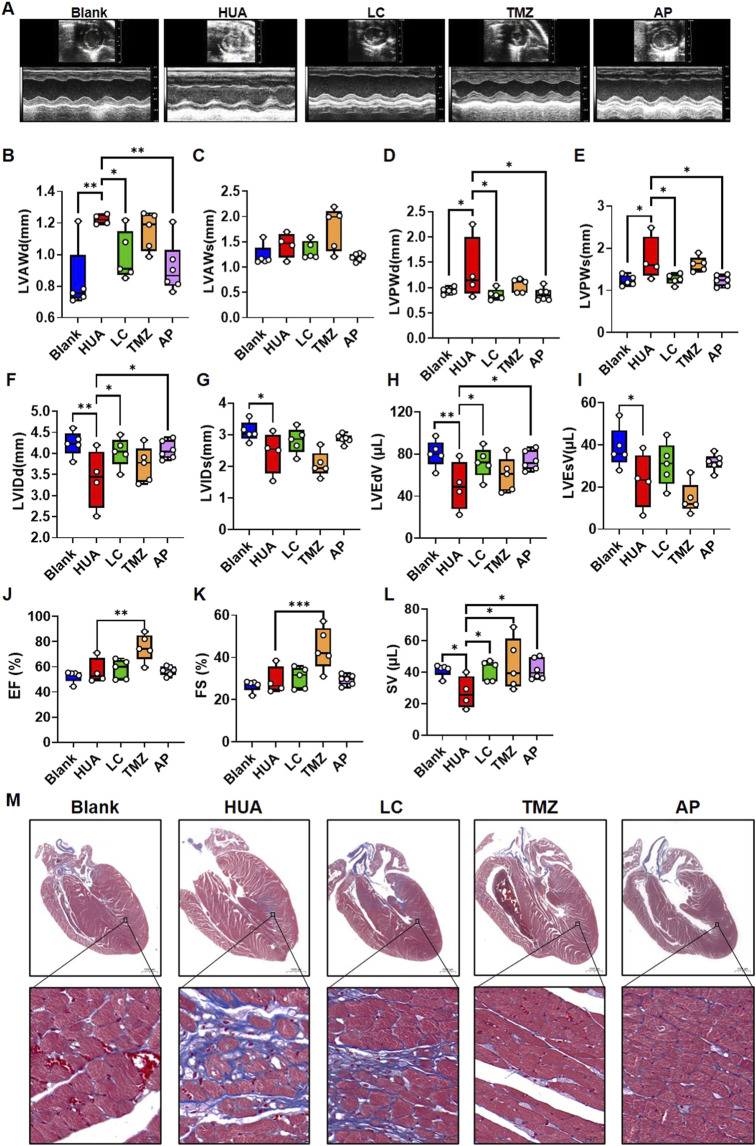
The influence of fatty acid metabolic agents’ intervention on the cardiac function of chronic HUA mice. **(A)** Representative photo of echocardiography examination in each group. Echocardiographic examination was performed after 8 weeks experiment. The summarized box plot of left ventricular anterior wall in diastolic-stage **(B)**, left ventricular anterior wall in systolic-stage **(C)**, left ventricular posterior wall in diastolic-stage **(D)**, left ventricular posterior wall in systolic-stage **(E)**, left ventricular internal dimension in diastolic-stage **(F)**, left ventricular internal dimension in systolic-stage **(G)**, left ventricular end-diastolic volume **(H)**, left ventricular end-systolic volume **(I)**, ejection fraction **(J)**, fractional shortening **(K)**, stroke volume **(L)**. blank *n* = 5, HUA *n* = 4, LC *n* = 5, TMZ *n* = 5, AP *n* = 6; **(M)** Masson staining of the heart samples in each group after8-weeks’ treatment. Red indicates muscle, blue indicates fibrous, scale bar = 1000 μm.

Throughout the experimental period, the plasma UA levels in the HUA, LC and TMZ groups remained significantly higher than those in the blank group and AP group (Supplementary Figure 6A). Compared with that of the blank mice, the body weight gain of the HUA mice was slower; LC and AP showed some ability to ameliorate this phenomenon, while TMZ nearly reversed the body weight changes (Supplementary Figure 6B). The echocardiography examination indicated that HUA mice showed increased left ventricular anterior and posterior wall thickness in the diastolic-stage, and posterior wall in the systolic-stage, while the thickening could be ameliorated by LC and AP intervention. TMZ showed no significant difference (Figures 6A–E). Correspondingly, left ventricular internal dimensions and end volume in the HUA group were decreased compare with those in the blank group, but LC intervention ameliorated the HUA-associated decrease in internal dimensions and end volume in diastolic-stage rather than systolic-stage, as did the urate-lowering agent AP, while TMZ did not show significant improvement (Figures 6F–I). However, TMZ intervention increased the ejection fraction and fractional shortening, and this phenomenon was not observed in the other groups (Figures 6J, K). Both LC and TMZ intervention ameliorated HUA-associated decreases in stroke volume, as did AP intervention (Figure 6L). Myocardial interstitial fibrosis is a common pathological change in LVR that can be described as abnormal deposition of collagenous fibers. Interstitial fibrosis was also observed in chronic HUA hearts in a previous study (Yan et al., 2018). Subsequently, we performed Masson staining to examine the effects of drugs targeting FA on HUA-associated interstitial fibrosis. Among the mice, two of the mice in the HUA group and two of the mice in the LC group exhibited myocardial interstitial fibrosis. Interstitial fibrosis was not observed in the TMZ group (Figure 6M; Supplementary Figure 6C).

Although *in vitro* experiment indicated TMZ showed exacerbate effect on UA induced cardiomyocytic injury, the *in vivo* experiment showed an opposite tendency. In cardiovascular system, LC and TMZ are not only influence cardiomyocytes but also may influence other systems’ working followed by indirectly feedback to cardiomyocytes. We examined plasma catecholamines by ELISA, which is also one of the risk factors contribute to LVR (Cohn, 2001). The result indicated that HUA didn’t influence plasma epinephrine, but increased noradrenaline to some extent, which could be rescued by TMZ and AP (Supplementary Figure 6D). This may partly explain the anti-fibrosis effect of TMZ.

## 4 Discussion

UA is the end product of endogenous purine metabolism. Under physiological conditions, the UA concentration in human blood is approximately 5.8 mg/dL in men and 4.5 mg/dL in women (Suzuki et al., 2013). HUA is an abnormally elevated blood UA concentration caused by a variety of triggers and can be diagnosed as blood UA ≥7 mg/dL in men and serum uric acid ≥6 mg/dL in women (Valsaraj et al., 2020; Zhang et al., 2020). In HUA, all organs are exposed to excessive UA in the intraorganellar environment. Metabolism abnormalities induced by exposure to excessive UA are important factors mediating the pathological development of HUA-associated multiple organ complications (Bergamini et al., 2009; Borghi et al., 2020). The cardiovascular risk and mortality in patients with gout can be reduced *via* UA excretion agent benzbromarone therapy (Kang et al., 2021). In a number of tissues, soluble uric acid has been shown to influence pathological development *via* regulating transforming growth factor β1, nicotinamide adenine dinucleotide phosphate oxidase and AMP-activated protein kinase mediated reactive oxygen species and inflammation (Madlala et al., 2016; Kimura et al., 2020). These mechanisms are all close related to metabolic disorders. In this study, we demonstrate that UA-induced cardiomyocyte FA metabolic dysfunction is the cause of HUA-associated LVR, and exogenous intervention with drugs targeting FA metabolism ameliorates HUA-associated left ventricular anterior wall thickening.

Energy metabolism plays pivotal roles in cellular physiological function, and its interplay with cardiac pathological processes has attracted broad interest. Currently, insufficient myocardial energy has been considered one of the mechanisms underlying the incidence and development of LVR (Xiong et al., 2019). Previously, scientists explored the impact of multiple risk factors on cardiomyocyte energy metabolism, such as sympathetic nervous excitation, type 2 diabetes, anticancer therapy and HUA (Desrois et al., 2014; Wu et al., 2016; Zhi et al., 2016; Li et al., 2020). It’s also reported that UA treatment of cardiomyocytes caused ATP levels to decrease, which is an indicator or energy insufficient (Gao et al., 2021). UA induced insulin resistance and further inhibited GLUT4-mediated glucose transmembrane transport to limit glucose uptake by cardiomyocytes (Zhi et al., 2016). However, the energy needed to sustain cardiomyocyte physiological activity is supplied by both glucose and FA metabolism, although FA oxidation rather than glucose metabolism is the predominant energy source (Lopaschuk et al., 2010). Exogenous UA exposure induced cardiomyocyte lipid deposition in a dose-dependent manner. Excessive cytoplasmic lipid accumulation in cardiomyocytes leads to cellular lipotoxicity and thereby promotes a series of harmful conditions, such as insulin resistance and oxidative stress (Jia et al., 2016; Li et al., 2020). UA-induced lipid deposition could be attenuated by LC, which promotes FA mitochondrial elimination. Therefore, UA has the potential to induce cardiomyocyte FA metabolic dysfunction. It would be interesting to explore the mechanisms by which UA regulates FA metabolism and to determine their significance for pathological processes.

The metabolic link between FA and cardiomyocyte injury induced by a large number of risk factors has attracted much attention. For example, type 2 diabetes increases FA uptake and oxidation in the heart (Rijzewijk et al., 2009), high salt-induced cardiac hypertrophy was associated with no changes in FA metabolism (Grover-McKay et al., 1989), and dilated cardiomyopathy was associated with decreased FA oxidation (Dávila-Román et al., 2002). UA induced cardiomyocyte cytoplasmic lipid deposition, and which step dominates this process is worth exploring. FA metabolism can be separated into four steps: membrane transporter-mediated FA uptake, transport from the cytoplasm to mitochondria, β-oxidation and acetyl-CoA synthesis, and the TAC cycle. Regarding UA-induced changes in the transcriptional landscape, *FABP3*, which mediates FA transmembrane trafficking, was downregulated, while *CPT1*, which mediates mitochondrial transport and β-oxidation, was upregulated. This observation indicates that cardiomyocytes triggered self-compensation through reduced exogenous FA uptake and increased intracellular metabolism in response to excessive lipids in the cytoplasm. CPT1 has three isoforms, CPT1A, CPT1B, and CPT1C. CPT1A is expressed in the liver, kidney, fibroblasts, and heart; CPT1B is expressed in skeletal muscle, the heart, and brown and white adipocytes; and CPT1C is expressed in the brain (Wang et al., 1998). The RNA-seq data showed significant differences for *CPT1A* and *CPT1C* rather than *CPT1B*, and we speculate that this may have been because these two isoforms showed lower abundance in cardiomyocytes and were thereby more sensitive. A more precise examination of *CPT1A* and *CPT1B* transcript expression was performed by qPCR and revealed the significant upregulation of *CPT1A* and *CPT1B*. However, western blot analysis showed that only CPT1B was suppressed at the protein level by UA stimulation. CPT1B is the rate-limiting enzyme in cardiomyocytes that mediates FA transport into mitochondria for oxidation (Wang et al., 2020; Angelini et al., 2021). Therefore, our results suggest that UA may induce FA metabolic disorders *via* CPT1B-mediated mitochondrial transport.

Most FAs in cells are esterified to glycerol or are components of phospholipids and sphingolipids (Shevchenko and Simons, 2010). The imbalance of FA metabolism often disrupts cellular lipid homeostasis and promotes a variety of disease states. UA increased the levels of cardiomyocytic glycerolipids, while the levels of phospholipids and sphingolipids decreased. Glycerolipids contain Triglyceride (TG) diacylglycerol (DG). TG are the primary source of energy-producing FA, and the increased levels of TG are a marker of general cellular lipid overload (Listenberger et al., 2003; Drosatos et al., 2011). DG can function as a second messenger to mediate cellular signaling. DG overload is also the cause of insulin resistance and endoplasmic reticulum stress (Erion and Shulman, 2010; Akoumi et al., 2017). UA increased cardiomyocyte glycerolipids, particularly 16:0 and 18:01 in TGs. 16:0 FA, is also known as palmitic acid. Palmitic acid is a well-known risk factors of cardiomyocytic apoptosis and cardiac hypertrophy (Zhao et al., 2016; Adrian et al., 2017; Wang et al., 2021). In previously publications, palmitic acid was associated with increased transcriptional level of CPT1 and decreased protein level of CPT1, which is consistent with our results (Sieber et al., 2013; Wen et al., 2017). 18:01 FA, is also known as oleic acid. In most current researches, oleic acid is a protective molecule for cardiomyocytes (Al-Shudiefat et al., 2013; Samanta et al., 2020). Oleic acid was also associated with increased transcriptional level of CPT1 (Kim et al., 2013), which is consistent with our results, but the impact on CPT1 protein level has not been reported. Considerate the opposite effect of palmitic acid and oleic acid on cardiomyocytes, we speculate the degree of UA induced cardiomyocytic injury is associated with the balance of increased palmitic acid and oleic acid. In condition of HUA, limited-consumption of palmitic acid-rich food may help delayed cardiac pathological development.

FA metabolic dysfunction is a contributing factor to LVR, and long-term LVR creates a vicious cycle that exacerbates the development of heart failure and leads to a poor prognosis in other cardiovascular diseases (Lionetti et al., 2011; Barbieri et al., 2019). Consistent with the observations in UA short-term stimulated cardiomyocytes, there was a trend toward reduced CPT1B levels in the heart tissues of HUA mice compared with the blank control mice. Long-term CPT1B inhibition can cause lipotoxicity in the heart under pathological stress, leading to more cardiomyocyte apoptosis and exacerbation of cardiac hypertrophy (He et al., 2012; Haynie et al., 2014). Epidemiological data have shown that serum uric acid levels are associated with cardiac hypertrophy in patients with underlying risk factors, such as renal transplant, non-valvular atrial fibrillation, metabolic syndrome and menopause (Caliskan et al., 2011; Yu et al., 2015; Yu et al., 2015; Liang et al., 2016). Consistently, our data also show that long-term HUA induced LVR in mice, with a thickened ventricular wall and decreased internal diameter. These results suggest that CPT1B inhibition-mediated FA metabolic dysfunction is critical for HUA-associated LVR. In addition, the performance of CPT1B showed great individual differences in long-term treatment of both blank and model group. Some researchers proposed that UA may act on heart in an early unstable stage of the disease, in the stable stages other factors may overshadow its effects (Maloberti et al., 2021).

Optimization of the strategies used for pharmacological intervention based on energy metabolic disorder is the focus of clinical pharmacology. The drugs that affect cellular energy metabolism can currently be classified into three types: the first class regulates the utilization of energy substrates, such as LC and TMZ; the second class increases mitochondrial energy conversion efficacy and includes coenzyme Q10; and the third class involves direct supplementation of high-energy phosphate compounds, such as phosphocreatine. In this study, FA-to-mitochondrial transport was the key to HUA-associated LVR. Therefore, two drugs, LC and TMZ, were used to assess therapeutic effects on HUA-associated LVR. LC is an adjuvant that to facilitates CPT1 mediate FA transport to the mitochondrial matrix for β-oxidation (Wu et al., 2017). TMZ can inhibit mitochondrial long-chain 3-ketoacyl coenzyme A thiolase and therefore shows an anti-β-oxidation effect (Marazzi et al., 2007). LC exerted a protective effect by attenuating HUA-induced changes in left ventricular wall thickness, while TMZ did not induce any improvement. However, although long-term LC treatment improved HUA-associated LVR, myocardial interstitial fibrosis was also observed in LC-treated mice, but the fibrosis can be rescued by TMZ as well as AP. We noted that HUA may increase noradrenaline to some extent, and could be rescued by TMZ and AP treatment. Noradrenaline is a well-known hormone closely associated with vasoconstrictor. Increased noradrenaline reveals potential decreased oxygen delivery to active tissues. FA oxidation requires more oxygen than glucose oxidation, and LC stimulates FA oxidation accompany with increasing oxygen consumption (Mollica et al., 2001). Hypoxia is a risk factor that stimulates cardiac fibroblast production and therefore leads to interstitial fibrosis (Wang et al., 2016). Therefore, we speculate that the interstitial fibrosis observed in HUA mice is mainly correlated with noradrenaline associated vascular effect. From the angle of cardiomyocytes injury, LC intervention is an effective solution to ameliorate HUA-associated LVR.

Some limitations of our study should be mentioned. HUA was diagnosed as UA ≥7 mg/dL in men and serum uric acid ≥6 mg/dL in women. To better observe the influence of UA on cardiomyocytes and LVR, we used a UA concentration of 15 mg/dL in *in vitro* experiments and a plasma UA concentration of 10–20 mg/dL in i*n vivo* experiments. This concentration of UA occurs in severe hyperuricemia and does not reflect the conditions in mild cases. Chronic hyperuricemia always accompanied with multiple complications such as kidney injury, non-alcoholic fatty liver and metabolic syndrome. We could not make sure the pathological development of LVR caused from UA directly injury or these complications indirectly influence. In summary, this study revealed that dysfunction of cellular transport of cytoplasmic FA to mitochondria plays a critical role in UA-induced cardiomyocyte injury and thereby promotes LVR development and identified CPT1B as the key factor influenced by UA. HUA-associated left ventricular wall thickening can be ameliorated by the FA oxidation-promoting agent LC. This study provides a novel perspective to improve our understanding of the mechanism of HUA-associated LVR and provides important information for pharmacological intervention. However, although we have shown the importance of FA metabolism in HUA-associated LVR pathological development, how it influences heart diseases and how to optimize metabolism-targeted therapeutic strategies need to be further investigated.

## Data Availability

The datasets presented in this study can be found in online repositories. The names of the repository/repositories and accession number(s) can be found in the article/Supplementary Material.
