# Supplementary material for: L-carnitine attenuated hyperuricemia-associated left ventricular remodeling through ameliorating cardiomyocytic lipid deposition
Source: Front Pharmacol. 2023 Jan 31;14:1016633. doi: 10.3389/fphar.2023.1016633 (PMC9929955; doi:10.3389/fphar.2023.1016633)
Supplement: Supplementary file 1 [file DataSheet1.docx]

Supplementary Material


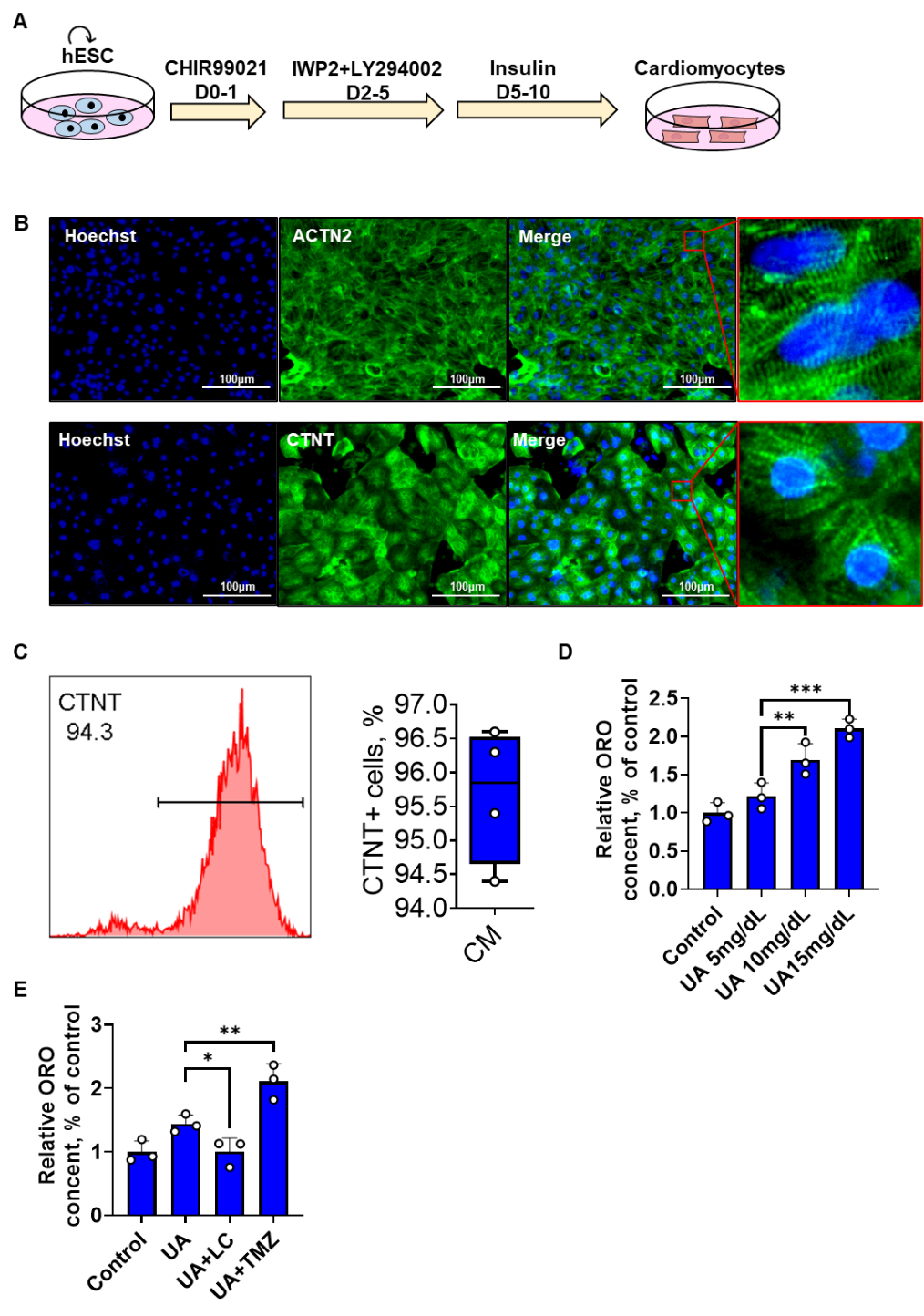


**Supplementary Figure 1.** Cardiomyocytes derived from hESC differentiation, related to Materials and Methods.

A. Diagram of cardiomyocytes differentiation strategies from hESC; B. Immunostaining of hESC-CM showed well-organized cardiac structure, scale bar=100μm; C. Flowcytometric analysis of CTNT showing the purity of generated cardiomyocytes (n=4); D. The quantification of Figure 1B; E. The quantification of Figure 1E.


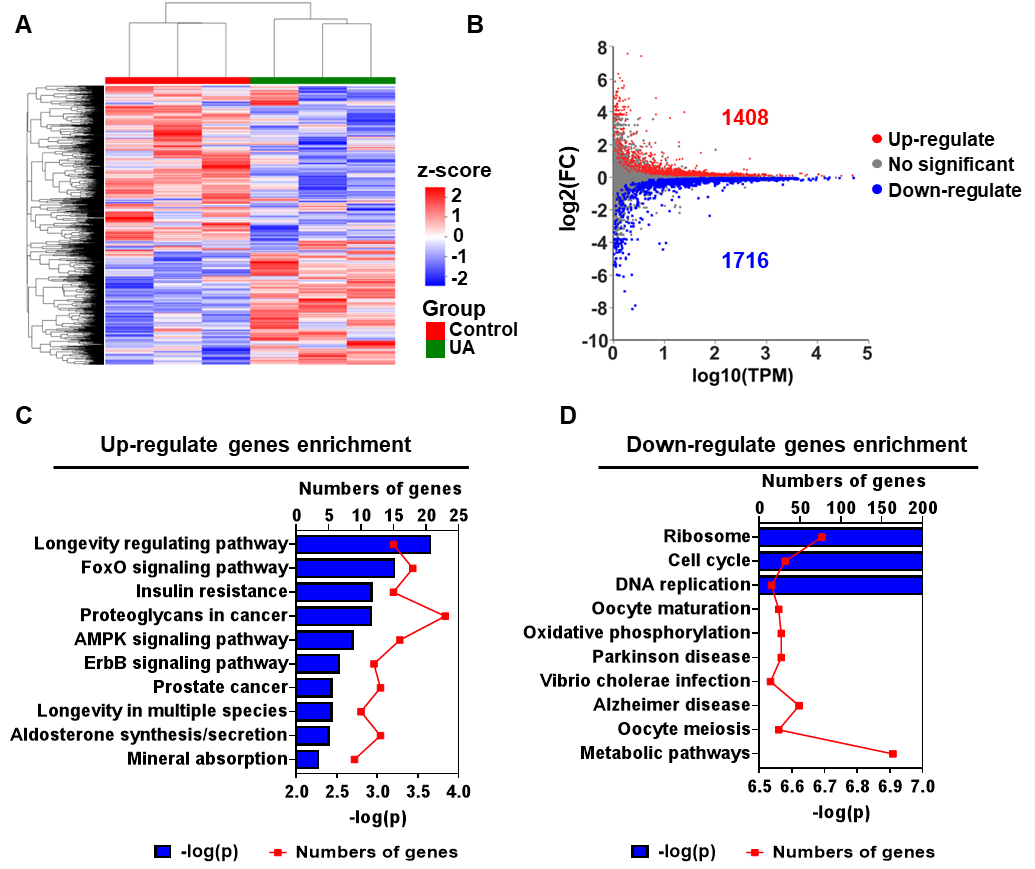


**Supplementary Figure 2.** Genes enrichment of UA treated cardiomyocytes, related to Figure 2.

A. Cluster of global gene expression profiles from RNAseq analysis; B. MA plot showing global gene folds change by UA stimulation, p＜0.05 was considered as significance; C. KEGG enrichment for the upregulated genes of UA treatment compared to control cells; D. KEGG enrichment for the downregulated genes of UA treatment compared to control cells.


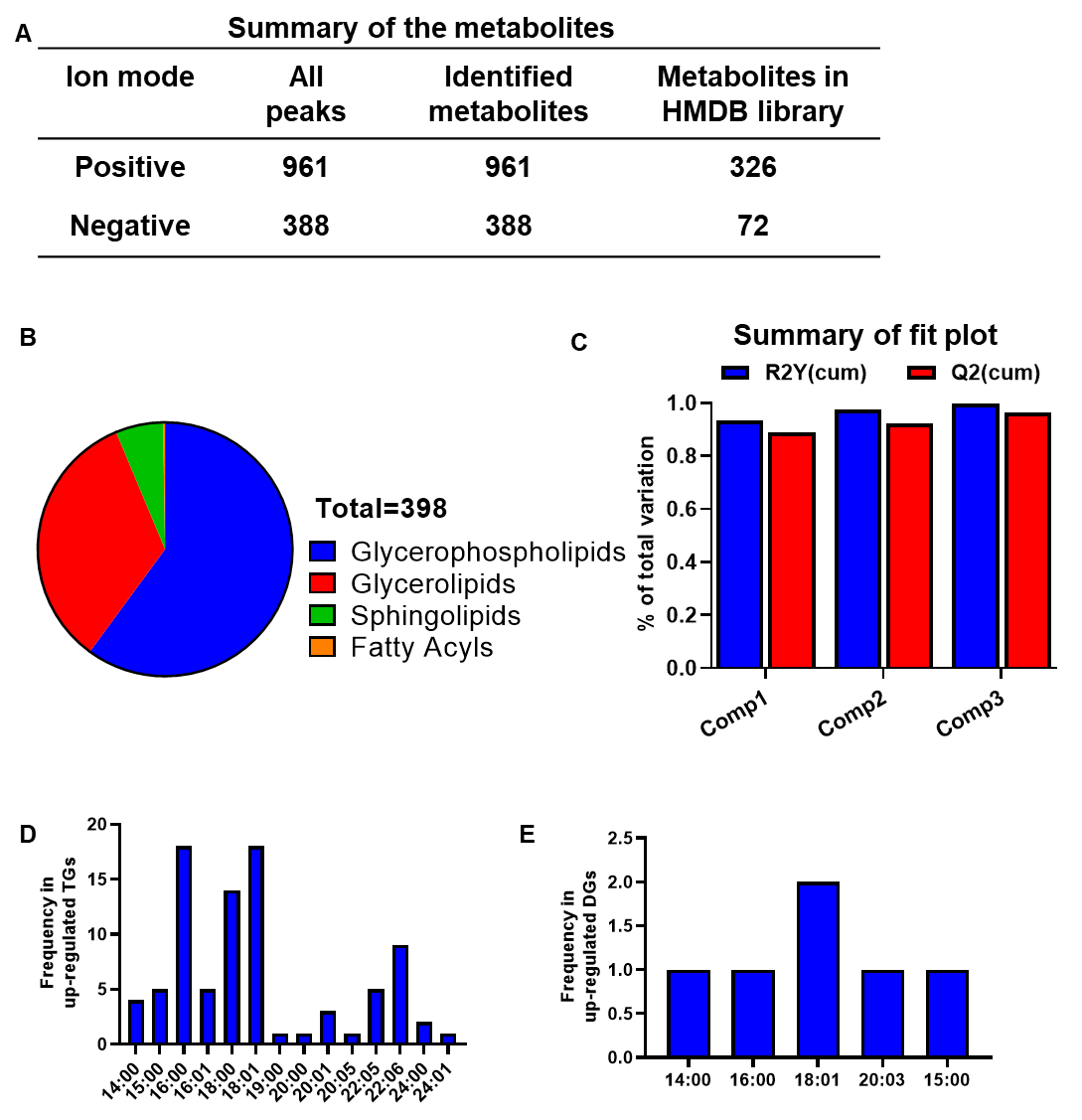


**Supplementary Figure 3.** basic information of cardiomyocytes lipidomic analysis, related to Figure 3.

A. The table summarized the detectable metabolites; B. The pie chart classified the metabolites into four groups; C. The summary of fit plot reflects the goodness of the model; D. The frequency of FA in up-regulated TGs; E. The frequency of FA in up-regulated DGs.


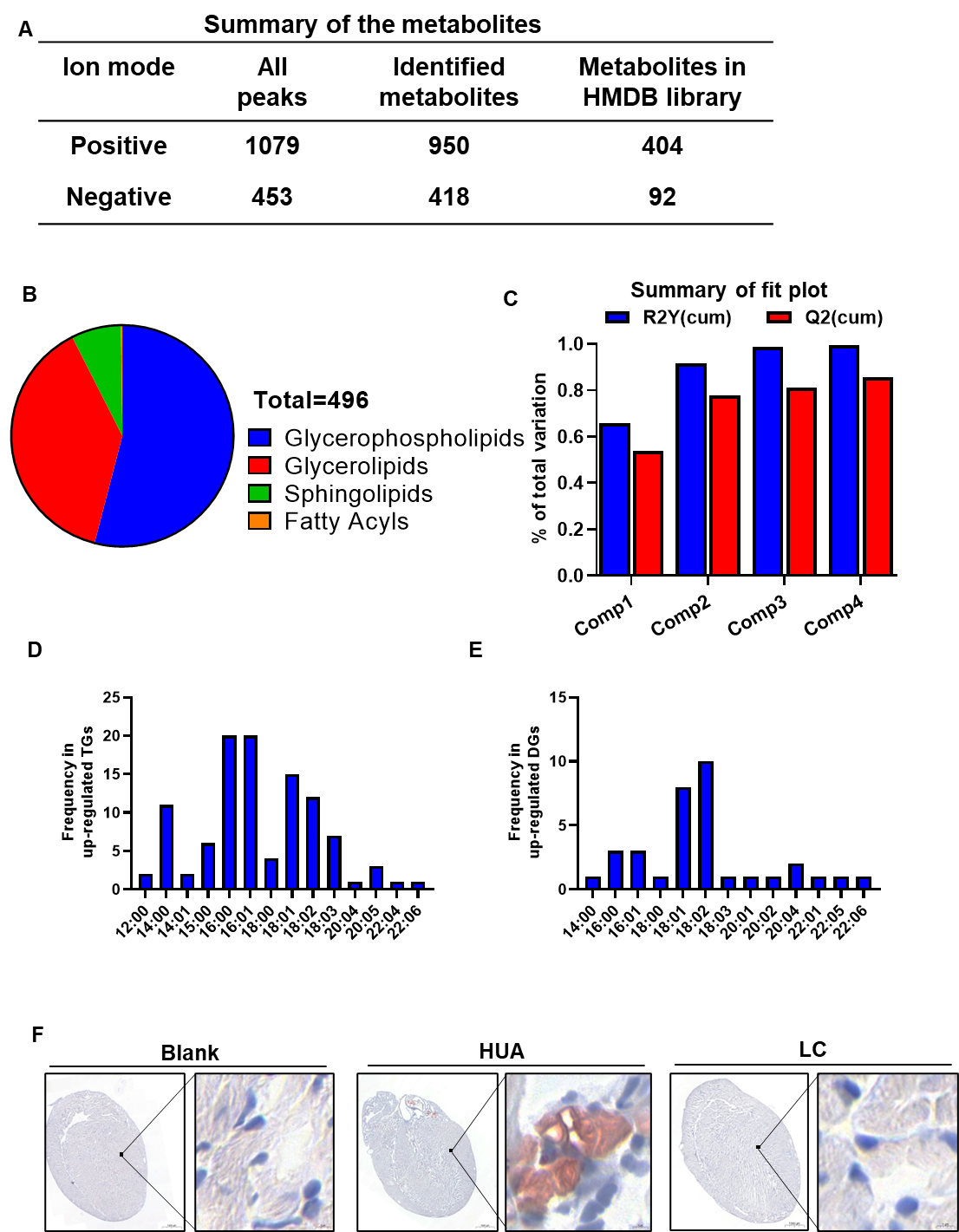


**Supplementary Figure 4.** basic information of heart tissues lipidomic analysis, related to Figure 4.

A. The table summarized the detectable metabolites; B. The pie chart classified the metabolites into four groups; C. The summary of fit plot reflects the goodness of the model; D. The frequency of FA in up-regulated TGs; E. The frequency of FA in up-regulated DGs; F. Oil-red O staining of hearts.


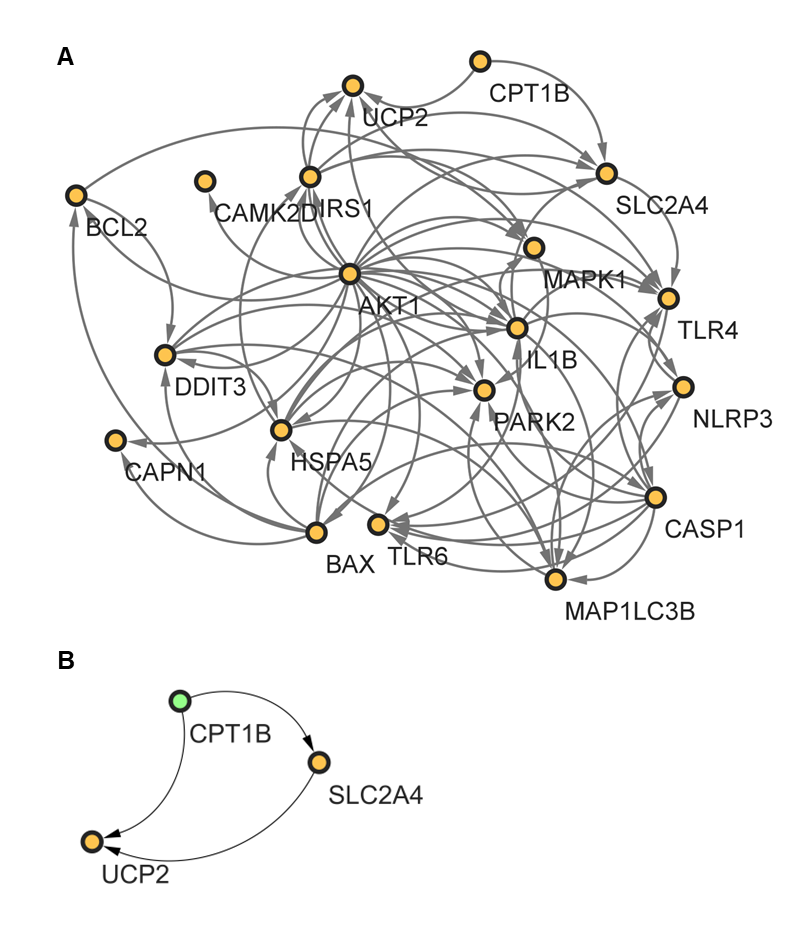


**Supplementary Figure 5.** Protein-protein interaction network, related to Figure 5.

A. The protein-protein interaction network between CPT1B and published UA associated mechanism; B. The interaction of the CPT1B associated first-stage nodes.


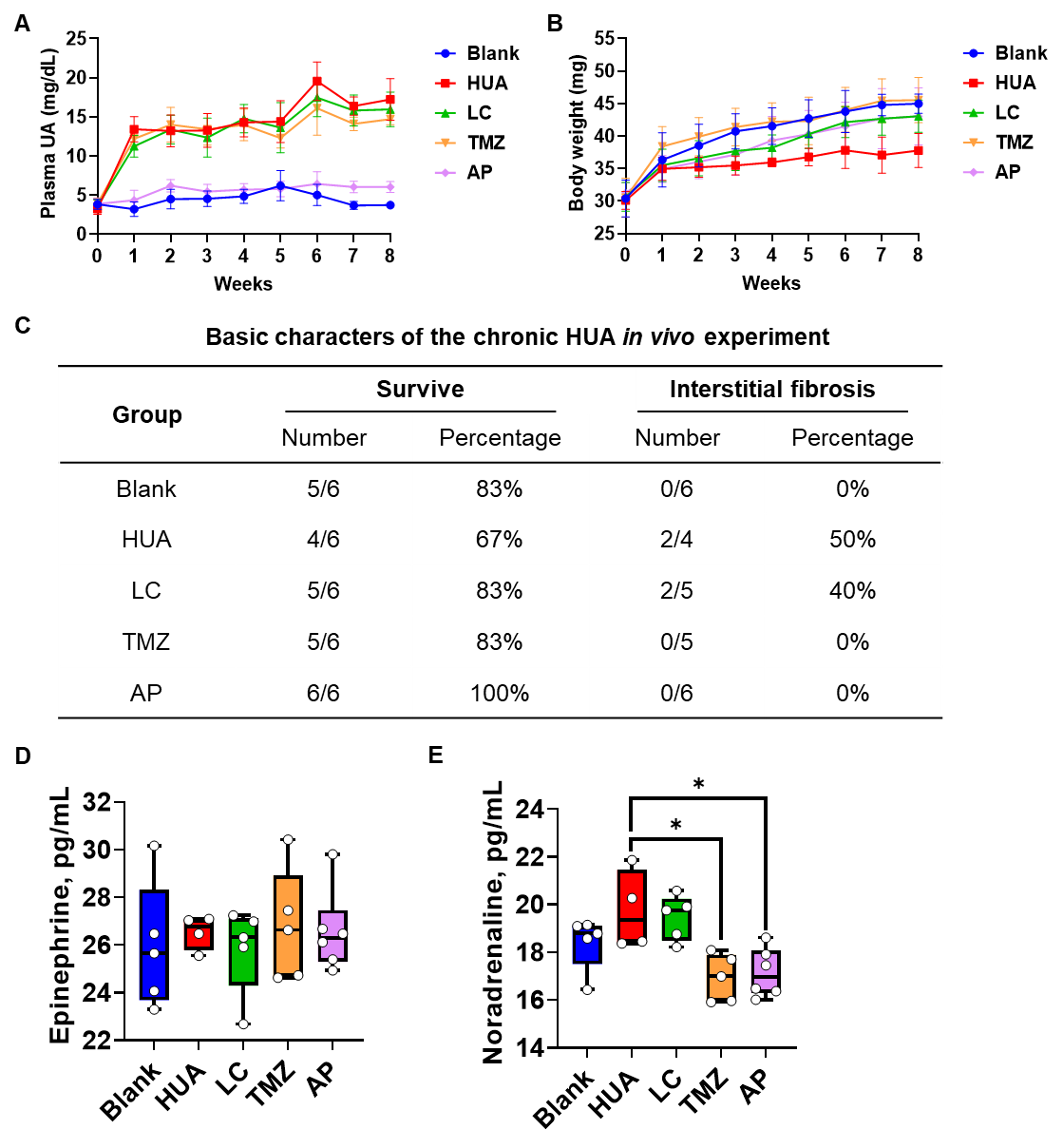


**Supplementary Figure 6.** basic information of chronic HUA *in vivo* experiment, related to Figure 6 and 6.

A. The time-course of plasma UA in each group; B. The time-course of body weight in each group; C. The summary of basic characters of the chronic HUA *in vivo* experiment in final; D. The plasma epinephrine concentrations; E. The plasma noradrenaline concentrations.

**Supplementary Table 1** List of primers used for real-time qPCR

| Gene | Sequence (5'-3') | |
| --- | --- | --- |
| *hFABP3* | Forward | GGCACCTGGAAGCTAGTGG |
|  | Reverse | CTGCCTGGTAGCAAAACCC |
| *hCPT1A* | Forward | TCCAGTTGGCTTATCGTGGTG |
|  | Reverse | TCCAGAGTCCGATTGATTTTTGC |
| *hCPT1B* | Forward | CATGTATCGCCGTAAACTGGAC |
|  | Reverse | TGGTAGGAGCACATAGGCACT |
| *hACSL3* | Forward | AGGAGGTCCAGCCATTGTTC |
|  | Reverse | CTATGAGGTTGGTTTTCCATGCT |
| *hACADM* | Forward | TGGATAACCAACGGAGGAAAAG |
|  | Reverse | CTGGGGTATCTGCTTCCACA |
| *hCS* | Forward | TGCTTCCTCCACGAATTTGAAA |
|  | Reverse | CCACCATACATCATGTCCACAG |
| *hACTB* | Forward | CATGTACGTTGCTATCCAGGC |
|  | Reverse | CTCCTTAATGTCACGCACGAT |
| *mCPT1B* | Forward | GACTTCCGGCTTAGTCGGG |
|  | Reverse | GAATAAGGCGTTTCTTCCAGGA |
| *mACTB* | Forward | CAGGCATTGCTGACAGGATG |
|  | Reverse | TGCTGATCCACATCTGCTGG |

**Supplementary Table 2** List of antibodies used in western blot and immunofluorescence

| Antibodies | Source | Identifier |
| --- | --- | --- |
| ACTN2 Polyclonal antibody | Proteintech | Cat No: 14221-1-AP; RRID: AB_2221547 |
| CTNT Polyclonal antibody | Proteintech | Cat No: 15513-1-AP; RRID: AB_2206563 |
| FABP3 Monoclonal antibody | Proteintech | Cat No: 60280-1-Ig; RRID: AB_2881398 |
| CPT1A Monoclonal antibody | Proteintech | Cat No: 66039-1-Ig; RRID: AB_11041710 |
| CPT1B Polyclonal antibody | Proteintech | Cat No: 22170-1-AP; RRID: AB_2713959 |
| β-Actin (4D3) monoclonal antibody | Bioworlde | Cat No: BS6007M; RRID: AB_2904238 |
| CoraLite488-conjugated Goat Anti-Rabbit IgG(H+L) | Proteintech | Cat No: SA00013-2; RRID: AB_2797132 |
| FITC conjugated Goat Anti-Mouse IgG (H+L) | Servicebio | Cat No: GB22301 |
| Cy3 conjugated Goat Anti-Rabbit IgG (H+L) | Servicebio | Cat No: GB21303; RRID: AB_2861435 |
| Peroxidase AffiniPure Goat Anti-Mouse IgG (H+L) | Jackson | Cat No: 115-035-146; RRID: AB_2307392 |
| Peroxidase AffiniPure Goat Anti-Rabbit IgG (H+L) | Jackson | Cat No: 111-035-144; RRID: AB_2307391 |

**Supplementary Table 3** List of discriminant metabolites in UA stimulated cardiomyocytes

| Metabolites | VIP value | -logP | Fold change | UA vs Control |
| --- | --- | --- | --- | --- |
| TG(18:1/18:1/22:5) | 1.571 | 4.803 | 1.016 | Up |
| PE(14:0/22:6) | 1.539 | 3.773 | 0.961 | Down |
| TG(18:0/18:1/22:6) | 1.538 | 4.468 | 1.016 | Up |
| PC(16:1/18:1) | 1.533 | 4.018 | 0.975 | Down |
| CL(18:1/16:1/16:1/16:1) | 1.522 | 1.939 | 0.908 | Down |
| PS(18:1/22:5) | 1.502 | 2.535 | 0.966 | Down |
| PC(14:0/14:0) | 1.501 | 3.246 | 0.965 | Down |
| DG(15:0/18:1) | 1.500 | 3.073 | 1.059 | Up |
| PS(18:1/20:2) | 1.486 | 2.245 | 0.972 | Down |
| PI(16:0/16:1) | 1.483 | 2.034 | 0.971 | Down |
| PC(14:0/20:5) | 1.482 | 3.722 | 0.972 | Down |
| LPE(16:0) | 1.479 | 2.199 | 0.967 | Down |
| PS(16:0/18:1) | 1.478 | 2.580 | 0.970 | Down |
| PC(18:1/20:5) | 1.463 | 3.110 | 0.972 | Down |
| PE(14:0/20:5) | 1.461 | 4.165 | 0.954 | Down |
| PS(18:1/18:1) | 1.461 | 2.175 | 0.970 | Down |
| PC(16:0/20:5) | 1.458 | 3.677 | 0.967 | Down |
| PS(18:1/22:6) | 1.449 | 2.149 | 0.975 | Down |
| PC(18:1/18:1) | 1.447 | 3.088 | 0.984 | Down |
| CL(18:1/16:1/16:1/18:1) | 1.445 | 1.762 | 0.960 | Down |
| PC(18:1/18:2) | 1.444 | 2.600 | 0.985 | Down |
| PE(18:1/14:0) | 1.444 | 4.146 | 0.956 | Down |
| PS(18:1/20:3) | 1.441 | 2.114 | 0.975 | Down |
| PS(18:1/20:4) | 1.431 | 2.046 | 0.971 | Down |
| PG(16:1/18:1) | 1.421 | 2.042 | 0.976 | Down |
| PE(14:0/20:3) | 1.409 | 3.331 | 0.966 | Down |
| TG(16:0/16:1/18:1) | 1.397 | 1.975 | 1.008 | Up |
| PE(16:1/22:6) | 1.393 | 2.832 | 0.976 | Down |
| PE(18:1/18:3) | 1.393 | 2.832 | 0.976 | Down |
| PE(16:0/18:2) | 1.391 | 3.343 | 0.977 | Down |
| PE(16:0/20:5) | 1.388 | 3.321 | 0.977 | Down |
| TG(18:1/18:1/22:6) | 1.386 | 3.044 | 1.009 | Up |
| TG(18:1/20:5/22:5) | 1.386 | 3.044 | 1.009 | Up |
| LPE(22:6) | 1.385 | 1.716 | 0.978 | Down |
| PC(18:1/20:2) | 1.384 | 1.534 | 1.013 | Up |
| PC(22:2/22:6) | 1.381 | 1.940 | 0.986 | Down |
| PE(18:1/18:2) | 1.381 | 3.479 | 0.981 | Down |
| PE(18:1/20:5) | 1.381 | 3.479 | 0.981 | Down |
| PS(16:0/22:5) | 1.378 | 1.736 | 0.978 | Down |
| PC(15:0/16:0) | 1.378 | 3.276 | 0.974 | Down |
| PC(16:1/20:4) | 1.376 | 1.864 | 0.967 | Down |
| PC(20:0/22:4) | 1.366 | 1.587 | 0.975 | Down |
| TG(16:1/18:1/18:1) | 1.364 | 2.827 | 1.006 | Up |
| PC(16:1/14:0) | 1.357 | 1.751 | 0.966 | Down |
| SM(d18:1/16:0) | 1.354 | 1.440 | 0.989 | Down |
| TG(18:0/16:0/16:0) | 1.344 | 1.375 | 1.016 | Up |
| PC(18:0/20:5) | 1.343 | 1.725 | 1.017 | Up |
| PS(16:0/22:6) | 1.341 | 1.749 | 0.967 | Down |
| DG(16:0/14:0) | 1.339 | 1.520 | 1.034 | Up |
| PC(15:0/20:3) | 1.338 | 1.997 | 0.988 | Down |
| PC(20:0/20:4) | 1.338 | 1.445 | 0.969 | Down |
| PE(16:1/20:4) | 1.327 | 1.987 | 0.985 | Down |
| LPC(14:0) | 1.326 | 1.317 | 0.964 | Down |
| PG(18:1/22:6) | 1.326 | 1.824 | 0.983 | Down |
| PC(18:4/20:4) | 1.325 | 3.096 | 0.957 | Down |
| SM(d18:1/14:0) | 1.320 | 1.402 | 0.977 | Down |
| PS(20:3/20:3) | 1.319 | 1.352 | 1.017 | Up |
| TG(15:0/16:0/24:0) | 1.318 | 1.993 | 1.047 | Up |
| PE(16:1/18:1) | 1.317 | 2.445 | 0.980 | Down |
| SM(d18:1/23:1) | 1.317 | 1.680 | 0.974 | Down |
| PE(18:1/22:4) | 1.315 | 1.367 | 1.008 | Up |
| PE(18:1/20:3) | 1.308 | 2.035 | 0.991 | Down |
| PE(15:0/18:1) | 1.308 | 1.493 | 1.012 | Up |
| PI(18:0/22:6) | 1.308 | 1.423 | 0.980 | Down |
| SM(d18:1/16:1) | 1.303 | 1.324 | 0.982 | Down |
| SM(d18:1/22:0) | 1.297 | 1.475 | 0.967 | Down |
| TG(18:0/18:0/18:0) | 1.286 | 1.415 | 1.009 | Up |
| PC(18:0/20:1) | 1.279 | 1.393 | 0.967 | Down |
| TG(20:1/18:1/22:5) | 1.277 | 2.026 | 1.021 | Up |
| TG(16:0/14:0/16:0) | 1.272 | 1.348 | 1.013 | Up |
| PI(18:0/18:0) | 1.267 | 1.649 | 0.986 | Down |
| PC(16:0/16:0) | 1.267 | 2.101 | 0.968 | Down |
| PS(20:3/18:2) | 1.265 | 2.756 | 1.043 | Up |
| CL(20:4/16:1/16:1/18:1) | 1.263 | 1.851 | 0.982 | Down |
| PS(18:0/22:5) | 1.258 | 1.396 | 0.985 | Down |
| TG(18:0/16:0/18:1) | 1.257 | 1.410 | 1.011 | Up |
| TG(16:0/14:0/18:1) | 1.250 | 1.766 | 1.008 | Up |
| SM(d18:2/14:0) | 1.249 | 1.383 | 0.979 | Down |
| TG(15:0/16:0/16:1) | 1.249 | 1.878 | 1.014 | Up |
| DG(18:1/20:3) | 1.244 | 2.029 | 1.054 | Up |
| TG(20:1/18:1/22:6) | 1.239 | 2.443 | 1.013 | Up |
| PC(15:0/20:2) | 1.232 | 1.308 | 0.990 | Down |
| PE(18:0/20:2) | 1.232 | 1.308 | 0.990 | Down |
| PC(20:0/22:6) | 1.220 | 2.020 | 0.989 | Down |
| PI(18:0/22:4) | 1.216 | 1.384 | 0.984 | Down |
| DG(18:0/20:3) | 1.212 | 1.686 | 0.981 | Down |
| PE(18:1p/22:6) | 1.209 | 2.448 | 0.989 | Down |
| PE(16:0/14:0) | 1.208 | 2.559 | 0.977 | Down |
| PS(20:3/20:5) | 1.205 | 1.827 | 0.990 | Down |
| PE(18:0/16:0) | 1.199 | 1.943 | 0.994 | Down |
| PS(18:0/20:3) | 1.196 | 1.369 | 0.987 | Down |
| PS(18:0/20:1) | 1.181 | 1.370 | 0.981 | Down |
| PG(18:1/18:3) | 1.181 | 1.789 | 0.967 | Down |
| LPE(20:2) | 1.180 | 1.530 | 0.975 | Down |
| PS(16:0/16:1) | 1.179 | 1.942 | 0.956 | Down |
| TG(16:0/16:0/22:6) | 1.177 | 2.066 | 1.013 | Up |
| PS(18:0/22:6) | 1.173 | 1.340 | 0.988 | Down |
| TG(18:0/16:0/18:0) | 1.173 | 2.223 | 1.013 | Up |
| TG(16:0/16:1/16:1) | 1.170 | 2.243 | 1.036 | Up |
| TG(18:0/16:0/19:0) | 1.168 | 2.065 | 1.027 | Up |
| PE(18:0/18:0) | 1.165 | 1.406 | 0.991 | Down |
| PE(18:0/20:3) | 1.165 | 1.406 | 0.991 | Down |
| TG(16:0/18:1/22:6) | 1.152 | 1.808 | 1.009 | Up |
| TG(18:0/18:1/20:0) | 1.139 | 1.690 | 1.012 | Up |
| PC(18:2/22:6) | 1.131 | 2.262 | 0.977 | Down |
| PE(16:1/20:5) | 1.130 | 1.797 | 0.976 | Down |
| TG(15:0/14:0/14:0) | 1.125 | 1.451 | 1.027 | Up |
| PE(16:1/16:1) | 1.119 | 1.424 | 0.983 | Down |
| TG(18:1/22:6/22:6) | 1.119 | 2.124 | 1.021 | Up |
| TG(18:0/16:0/20:1) | 1.116 | 1.781 | 1.015 | Up |
| TG(15:0/16:0/22:6) | 1.108 | 1.725 | 1.054 | Up |
| TG(15:0/18:1/22:5) | 1.103 | 1.840 | 1.022 | Up |
| PE(18:0/18:1) | 1.090 | 2.179 | 0.993 | Down |
| PG(22:6/22:6) | 1.089 | 1.812 | 0.985 | Down |
| Cer(d18:1/14:0) | 1.087 | 1.626 | 0.985 | Down |
| PC(18:4/18:1) | 1.086 | 1.656 | 0.933 | Down |
| PE(18:2/22:6) | 1.066 | 1.351 | 0.986 | Down |
| TG(18:0/18:1/24:0) | 1.062 | 1.679 | 1.048 | Up |
| PE(20:3/18:2) | 1.060 | 1.372 | 0.986 | Down |
| TG(18:0/16:0/24:1) | 1.046 | 1.550 | 1.027 | Up |
| TG(18:0/22:5/22:6) | 1.033 | 1.741 | 1.026 | Up |
| PC(20:1/20:1) | 1.031 | 1.448 | 0.934 | Down |
| PC(20:1/20:4) | 1.018 | 1.464 | 0.994 | Down |

**Supplementary Table 4** List of discriminant metabolites in HUA mice heart

| Metabolites | VIP value | -logP | Fold change | UA vs Control |
| --- | --- | --- | --- | --- |
| PE(18:2/22:6) | 1.888 | 3.070 | 1.020 | Up |
| PE(20:5/22:6) | 1.888 | 3.070 | 1.020 | Up |
| PC(16:1/18:3) | 1.741 | 4.047 | 0.976 | Down |
| DG(18:2/18:2) | 1.697 | 4.315 | 1.062 | Up |
| DG(18:2/20:4) | 1.674 | 3.215 | 1.032 | Up |
| PG(22:5/22:6) | 1.673 | 2.262 | 0.978 | Down |
| PG(18:2/22:6) | 1.653 | 3.996 | 0.980 | Down |
| DG(18:1/22:5) | 1.646 | 2.123 | 1.045 | Up |
| DG(18:1/18:2) | 1.635 | 4.063 | 1.043 | Up |
| LPC(24:0) | 1.628 | 2.636 | 1.016 | Up |
| DG(20:2/18:2) | 1.592 | 3.419 | 1.041 | Up |
| Cer(d18:1/16:0) | 1.586 | 3.674 | 0.985 | Down |
| PE(18:3/22:6) | 1.584 | 3.194 | 1.023 | Up |
| DG(18:2/22:6) | 1.583 | 2.155 | 1.020 | Up |
| DG(18:1/20:4) | 1.576 | 2.831 | 1.019 | Up |
| LPC(20:5) | 1.572 | 4.019 | 0.964 | Down |
| PG(18:0/18:2) | 1.553 | 3.252 | 1.012 | Up |
| TG(18:0/18:0/22:4) | 1.539 | 2.404 | 1.044 | Up |
| PG(20:4/22:6) | 1.513 | 3.221 | 0.984 | Down |
| DG(18:3/18:2) | 1.505 | 3.522 | 1.102 | Up |
| DG(16:0/18:1) | 1.503 | 3.295 | 1.031 | Up |
| PG(16:0/18:2) | 1.499 | 3.487 | 0.973 | Down |
| PS(22:6/22:6) | 1.498 | 2.866 | 1.029 | Up |
| PC(18:4/20:4) | 1.498 | 3.385 | 0.976 | Down |
| PC(16:1/16:1) | 1.486 | 3.173 | 0.970 | Down |
| TG(16:1/16:1/20:4) | 1.482 | 2.929 | 1.048 | Up |
| TG(16:0/22:5/22:6) | 1.474 | 2.412 | 0.968 | Down |
| DG(16:0/18:2) | 1.446 | 3.198 | 1.030 | Up |
| TG(16:0/22:4/22:6) | 1.436 | 3.129 | 0.952 | Down |
| PG(18:1/22:6) | 1.436 | 3.105 | 0.980 | Down |
| PC(14:0/22:6) | 1.430 | 3.133 | 0.975 | Down |
| DG(20:1/18:1) | 1.426 | 2.207 | 1.045 | Up |
| PC(18:4/20:5) | 1.420 | 3.083 | 0.974 | Down |
| LPC(22:5) | 1.415 | 3.021 | 0.980 | Down |
| TG(18:1/22:5/22:6) | 1.412 | 2.027 | 0.969 | Down |
| PC(22:5/22:6) | 1.404 | 2.920 | 0.978 | Down |
| PC(18:0/20:4) | 1.401 | 2.971 | 0.987 | Down |
| PC(15:0/20:4) | 1.400 | 2.754 | 0.988 | Down |
| PE(18:3/20:4) | 1.399 | 2.868 | 1.030 | Up |
| TG(16:0/20:4/22:6) | 1.397 | 1.994 | 0.978 | Down |
| PC(15:0/18:2) | 1.392 | 2.805 | 0.983 | Down |
| PC(18:4/18:3) | 1.378 | 1.813 | 0.978 | Down |
| DG(18:1/18:1) | 1.372 | 2.827 | 0.974 | Down |
| TG(18:0/20:4/22:6) | 1.369 | 2.365 | 0.975 | Down |
| Cer(d18:1/23:0) | 1.364 | 2.311 | 0.973 | Down |
| Cer(d18:0/20:0) | 1.352 | 2.633 | 0.976 | Down |
| SM(d18:2/16:0) | 1.344 | 1.334 | 1.006 | Up |
| Cer(d17:1/18:0) | 1.341 | 2.491 | 0.968 | Down |
| LPG(18:1) | 1.331 | 2.543 | 0.978 | Down |
| PC(24:1/20:4) | 1.324 | 1.753 | 1.012 | Up |
| PC(16:1/22:4) | 1.324 | 2.585 | 1.016 | Up |
| DG(18:0/18:1) | 1.320 | 2.379 | 1.021 | Up |
| PC(15:0/20:2) | 1.316 | 2.502 | 0.985 | Down |
| PG(22:6/22:6) | 1.315 | 1.682 | 0.985 | Down |
| TG(18:0/22:4/22:6) | 1.313 | 2.530 | 0.928 | Down |
| cPA(18:0) | 1.310 | 1.499 | 0.970 | Down |
| DG(16:1/18:2) | 1.308 | 2.308 | 1.046 | Up |
| TG(18:0/20:4/22:4) | 1.303 | 2.206 | 0.973 | Down |
| PE(18:0/20:3) | 1.300 | 2.413 | 0.988 | Down |
| LPC(14:0) | 1.298 | 2.384 | 0.973 | Down |
| PE(18:2/18:2) | 1.292 | 1.827 | 1.025 | Up |
| LPI(18:2) | 1.290 | 1.797 | 0.979 | Down |
| LPC(15:0) | 1.289 | 2.241 | 0.968 | Down |
| PC(17:0/22:6) | 1.286 | 2.353 | 0.971 | Down |
| PG(18:2/20:4) | 1.285 | 2.237 | 0.981 | Down |
| TG(18:0/22:5/22:6) | 1.279 | 2.224 | 0.969 | Down |
| TG(16:1/18:1/18:2) | 1.272 | 2.214 | 1.039 | Up |
| DG(18:1/22:1) | 1.248 | 1.404 | 1.032 | Up |
| TG(15:0/12:0/14:0) | 1.240 | 2.088 | 1.115 | Up |
| PE(18:0/18:1) | 1.234 | 2.120 | 0.993 | Down |
| DG(16:1/18:1) | 1.228 | 1.935 | 1.047 | Up |
| PE(15:0/22:6) | 1.224 | 2.092 | 0.980 | Down |
| TG(18:0/22:4/22:5) | 1.223 | 2.118 | 0.964 | Down |
| PC(18:0/24:1) | 1.221 | 1.648 | 0.982 | Down |
| PE(18:2/20:4) | 1.218 | 1.415 | 1.012 | Up |
| Cer(d18:1/18:0) | 1.216 | 1.974 | 0.984 | Down |
| PC(18:1/22:6) | 1.215 | 1.927 | 0.989 | Down |
| TG(18:1/18:2/18:2) | 1.210 | 1.972 | 1.051 | Up |
| LPE(22:4) | 1.207 | 2.104 | 0.982 | Down |
| Cer(d18:2/18:0) | 1.203 | 1.871 | 0.986 | Down |
| TG(16:0/16:0/20:5) | 1.199 | 1.839 | 1.035 | Up |
| TG(16:0/16:0/18:3) | 1.199 | 1.684 | 1.041 | Up |
| DG(16:0/16:1) | 1.194 | 1.503 | 1.036 | Up |
| TG(15:0/16:0/18:1) | 1.192 | 1.838 | 1.038 | Up |
| Cer(d18:1/22:0) | 1.191 | 1.995 | 0.986 | Down |
| PE(16:0/20:3) | 1.189 | 1.881 | 0.989 | Down |
| TG(18:1/18:1/18:1) | 1.188 | 1.935 | 1.022 | Up |
| TG(16:1/16:1/20:5) | 1.184 | 1.432 | 1.026 | Up |
| PC(18:4/18:2) | 1.183 | 1.898 | 0.985 | Down |
| PE(20:1/18:1) | 1.177 | 1.971 | 1.018 | Up |
| TG(15:0/16:0/18:2) | 1.176 | 1.550 | 1.038 | Up |
| TG(16:1/14:1/18:3) | 1.176 | 1.931 | 1.005 | Up |
| PC(20:1/22:6) | 1.172 | 1.839 | 0.982 | Down |
| PC(15:0/16:0) | 1.169 | 1.815 | 0.989 | Down |
| DG(18:0/22:6) | 1.168 | 1.608 | 0.988 | Down |
| PE(18:1/18:2) | 1.161 | 1.778 | 1.015 | Up |
| PE(22:5/18:2) | 1.160 | 1.903 | 0.985 | Down |
| TG(16:1/18:2/18:3) | 1.151 | 1.406 | 1.050 | Up |
| PC(18:4/18:1) | 1.151 | 1.640 | 0.989 | Down |
| TG(16:1/18:2/18:2) | 1.151 | 1.481 | 1.047 | Up |
| PE(20:2/18:2) | 1.150 | 1.754 | 1.018 | Up |
| TG(16:1/18:1/18:1) | 1.148 | 1.535 | 1.024 | Up |
| LPI(18:1) | 1.146 | 1.450 | 0.979 | Down |
| PE(18:1/24:0) | 1.144 | 1.861 | 1.031 | Up |
| TG(16:1/18:2/22:6) | 1.143 | 1.778 | 1.033 | Up |
| TG(16:0/16:0/18:1) | 1.143 | 1.768 | 1.025 | Up |
| TG(16:0/18:2/18:3) | 1.143 | 1.422 | 1.040 | Up |
| TG(16:0/14:0/18:1) | 1.142 | 1.823 | 1.035 | Up |
| LPC(16:0) | 1.139 | 1.575 | 0.986 | Down |
| DG(14:0/18:2) | 1.138 | 1.417 | 1.030 | Up |
| TG(16:0/14:0/16:1) | 1.130 | 1.718 | 1.049 | Up |
| PE(16:0/18:2) | 1.127 | 1.784 | 0.990 | Down |
| TG(16:1/16:1/16:1) | 1.127 | 1.515 | 1.060 | Up |
| TG(15:0/16:0/18:3) | 1.124 | 1.607 | 1.052 | Up |
| PC(16:0/18:2) | 1.123 | 1.586 | 0.990 | Down |
| TG(18:1/22:4/22:6) | 1.122 | 1.722 | 0.954 | Down |
| PC(15:0/18:1) | 1.121 | 1.772 | 0.989 | Down |
| PG(16:1/18:2) | 1.116 | 1.745 | 1.023 | Up |
| Cer(d18:1/14:0) | 1.115 | 1.361 | 0.986 | Down |
| TG(16:0/14:0/18:3) | 1.113 | 1.302 | 1.045 | Up |
| TG(18:0/16:0/18:1) | 1.102 | 1.359 | 1.029 | Up |
| TG(16:0/14:0/16:0) | 1.097 | 1.453 | 1.021 | Up |
| TG(15:0/14:0/16:1) | 1.096 | 1.602 | 1.046 | Up |
| DG(18:0/18:0) | 1.096 | 1.421 | 0.990 | Down |
| TG(16:1/14:1/16:1) | 1.094 | 1.442 | 1.059 | Up |
| TG(14:0/18:2/18:3) | 1.094 | 1.455 | 1.054 | Up |
| PE(14:0/22:6) | 1.094 | 1.710 | 0.982 | Down |
| LPC(20:3) | 1.094 | 1.518 | 0.979 | Down |
| LPC(22:4) | 1.093 | 1.567 | 0.984 | Down |
| TG(18:0/18:1/18:1) | 1.088 | 1.392 | 1.030 | Up |
| PG(18:1/18:2) | 1.087 | 1.678 | 0.981 | Down |
| TG(16:0/12:0/14:0) | 1.085 | 1.519 | 1.066 | Up |
| PC(18:1/24:0) | 1.078 | 1.649 | 1.032 | Up |
| TG(16:0/18:1/18:1) | 1.075 | 1.531 | 1.023 | Up |
| PI(18:1/20:4) | 1.075 | 1.529 | 0.983 | Down |
| PC(15:0/20:5) | 1.075 | 1.543 | 0.986 | Down |
| PG(16:0/20:3) | 1.070 | 1.535 | 1.009 | Up |
| PE(18:0/20:4) | 1.068 | 1.648 | 0.994 | Down |
| LPC(20:2) | 1.066 | 1.575 | 0.978 | Down |
| TG(16:0/14:0/18:2) | 1.066 | 1.388 | 1.038 | Up |
| PE(16:0/18:1) | 1.058 | 1.505 | 0.992 | Down |
| LPE(22:5) | 1.057 | 1.447 | 0.978 | Down |
| PC(18:0/18:2) | 1.054 | 1.583 | 0.991 | Down |
| PC(18:0/22:5) | 1.054 | 1.478 | 0.982 | Down |
| LPE(22:6) | 1.038 | 1.487 | 0.990 | Down |
| TG(16:1/18:2/20:5) | 1.038 | 1.309 | 1.052 | Up |
| Cer(d18:1/20:0) | 1.036 | 1.424 | 0.988 | Down |
| Cer(d18:0/16:0) | 1.036 | 1.331 | 0.984 | Down |
| PE(18:1/22:1) | 1.034 | 1.495 | 1.016 | Up |
| PG(16:0/18:1) | 1.032 | 1.422 | 0.991 | Down |
| TG(16:0/14:0/14:0) | 1.025 | 1.424 | 1.041 | Up |
| TG(15:0/16:1/16:1) | 1.021 | 1.380 | 1.040 | Up |
| PC(14:0/22:5) | 1.020 | 1.489 | 0.987 | Down |
| PC(16:0/20:4) | 1.007 | 1.422 | 0.989 | Down |
| PC(16:0/20:5) | 1.005 | 1.469 | 0.985 | Down |
| PE(18:1/22:0) | 1.005 | 1.414 | 1.021 | Up |
